# Induced protein expression in *Leptospira* spp. and its application to CRISPR/Cas9 mutant generation

**DOI:** 10.1038/s41598-025-88633-w

**Published:** 2025-02-05

**Authors:** L. G. V. Fernandes, A. L. T. O. Nascimento, J. E. Nally

**Affiliations:** 1 Infectious Bacterial Diseases Research Unit, USDA Agricultural Research Service, National Animal Disease Center, Ames, IA USA; 2 Laboratório de Desenvolvimento de Vacinas, Instituto Butantan, São Paulo, Brazil

**Keywords:** IPTG, Mutagenesis, *Leptospira*, Knockdown, dCas9, Knockout, Cas9, Genetic engineering, Pathogenesis

## Abstract

Expanding the genetic toolkit for *Leptospira* spp. is a crucial step toward advancing our understanding of the biology and virulence of these atypical bacteria. Pathogenic *Leptospira* are responsible for over 1 million human leptospirosis cases annually and significantly impact domestic animals. Bovine leptospirosis causes substantial financial losses due to abortion, stillbirths, and suboptimal reproductive performance. The advent of the CRISPR/Cas9 system has marked a turning point in genetic manipulation, with applications across multiple *Leptospira* species. However, incorporating controlled protein expression into existing genetic tools could further expand their utility. We developed and demonstrated the functionality of IPTG-inducible heterologous protein expression in *Leptospira* spp. This system was applied for regulated expression of dead Cas9 (dCas9) to generate knockdown mutants, and Cas9 to produce knockout mutants by inducing double-strand breaks (DSB) into desired targets. IPTG-induced dCas9 expression enabled validation of essential genes and non-coding RNAs. Additionally, IPTG-controlled Cas9 expression combined with a constitutive non-homologous end-joining (NHEJ) system allowed for successful recovery of knockout mutants, even in the absence of IPTG. These newly controlled protein expression systems will advance studies on the basic biology and virulence of *Leptospira*, as well as facilitate knockout mutant generation for improved veterinary vaccines.

## Introduction

Pathogenic *Leptospira*spp. are the causative agent of leptospirosis^1–3^, a worldwide neglected zoonosis responsible for more than 1 million human cases and approximately 60,000 deaths annually^4^. Leptospires are versatile bacteria with genome plasticity^5^ capable of expressing a distinct arsenal of proteins in response to environmental and host cues, including osmolarity, temperature and serum^6,7^^,^^8–11^. These bacteria can survive in wet environments and infect mammalian hosts through penetration of abraded skin or mucosa, rapidly disseminating in the bloodstream, where they overcome several host immune defenses, reaching immune privileged target organs^12^.

Recent advances in functional genomics^13–15^, plasmid delivery by *E. coli *conjugation^16^ and mutagenesis tools for *Leptospira *spp.^17–19^, are enabling the elucidation of the biology of *Leptospira*, and their pathophysiology and virulence factors. The generation of mutants is a pivotal step to confirm the roles of expressed genes, either by in vitro assays or evaluation of virulence in animal models^20–23^. In past years, the application of the “memory immunity” bacterial CRISPR/Cas9 system from *Streptococcus pyogenes* has advanced the generation of knockdown and knockout mutants in several species and serovars of *Leptospira*^18,19,^^24–26^.

The CRISPR/Cas9 system relies on the expression of a programmable RNA-guided nuclease, called Cas9, and a single-guide RNA (sgRNA), comprising a 5′ 20-nt sequence termed protospacer, that dictates Cas9 targets by base-pairing, and a scaffold region responsible for Cas9 binding^27,28^. Upon recognition of the protospacer adjacent motif (PAM) 5’ NGG 3’ and sgRNA:DNA base pairing, Cas9 utilizes its two nuclease domains to promote double-strand breaks (DSBs) in the target sequence, which needs to be repaired so cells can retain their viability^29,30^. Even though eukaryotes present with multi-protein non-homologous end-joining (NHEJ) pathways to re-ligate DNA ends in the absence of a template for recombination^31^, most bacterial cells lack such machinery, with a few exceptions^32^.

It was experimentally demonstrated for *Leptospira *spp. that DSBs caused by Cas9 are lethal to the cells^17,24^, and the first approach to overcome this obstacle was to employ a catalytically inactive Cas9 (dCas9). Mechanistically, target recognition by dCas9 is similar to that of Cas9, however, dCas9 is not capable of cutting the DNA, and acts merely as a steric barrier preventing RNA polymerase elongation^33^. This physical hindrance results in gene silencing, in a strategy termed CRISPR interference (CRISPRi), which was successfully used to generate several mutants in *Leptospira *spp.^21^^,^^34,35^^,^^26^. In its current state, CRISPRi with constitutively expressed dCas9 cannot differentiate a failed conjugation experiment from a lethality caused by silencing of an essential gene.

Lethal DSBs caused by Cas9 were overcome by co-expression of this nuclease with a prokaryotic version of the NHEJ from *Mycobacteria smegmatis*, composed of sequences coding for two proteins, LigD and Ku. This strategy allowed the recovery of knockout markerless mutants with target specificity but variable deletion lengths from the Cas9 cutting site^24^, since NHEJ is an error prone process. The success of the Cas9-NHEJ approach is dependent on the efficiency of conjugation, which tends to be low for certain species and serovars of *Leptospira*, and on the efficiency of DSB repair. At present, these efficiencies are interdependent events resulting in low mutant recovery rates for pathogenic *Leptospira*. Nonetheless, mutant recovery by Cas9-NHEJ appears to be higher than allelic exchange^20^, which was, for several years, the only technique available for targeted mutagenesis in *Leptospira*.

It is noteworthy that a system for controlled protein expression would open new horizons for both CRISPRi and Cas9-NHEJ methodologies, expanding the toolbox for *Leptospira* mutagenesis. In the case of CRISPRi, an inducible dCas9 expression would favor the validation of essential genes, as the same conjugation reaction could be evaluated in both the presence and absence of an inducer for dCas9 expression. For Cas9-NHEJ, repressed Cas9 expression would be beneficial for colony recovery since hypothetically, the frequency of this first step would be determined by the efficiency of conjugation. After colony recovery and growth of leptospires to high cell densities, Cas9 can then be expressed by the addition of an inducer; thus, the frequency of DNA repair by the NHEJ proteins would be acting in this subsequent step only, and thus increasing the chances of knockout mutant recovery.

In this study, we developed a cassette for heterologous protein expression in *Leptospira* spp. based on the lactose operon from *E. coli*. Protein expression induced by IPTG was demonstrated and the inducible cassette was used to control expression of dCas9 and Cas9 in *Leptospira*. The applicability of inducible expression of dCas9 for validating essential genes, and Cas9 for improved knockout mutant recovery were evaluated in distinct species and serovars of *Leptospira*.

## Methods

### Bacterial strains and media

Pathogenic *L. interrogans *serovar Copenhageni strain FIOCRUZ L1-130^36^ and serovar Canicola strain LAD-1^25 ^were grown in HAN medium^37^ at 29 °C. Saprophytic *L. biflexa* serovar Patoc strain Patoc1 was cultured in EMJH medium (Difco, BD, Franklin Lakes, NJ) or HAN medium, according to the experimental design. For agar plates, media was supplemented with 1.2% noble agar (Difco). To select for recombinant cells, spectinomycin was added at 40 µg/mL. *E. coli *strain β2163^38^, auxotrophic for diaminopimelic acid (DAP), was grown in Lysogeny Broth (LB, Difco) medium supplemented with DAP (0.3 mM, Sigma).

### Development of an IPTG-inducible cassette for *Leptospira* spp

**Fig. 1:**
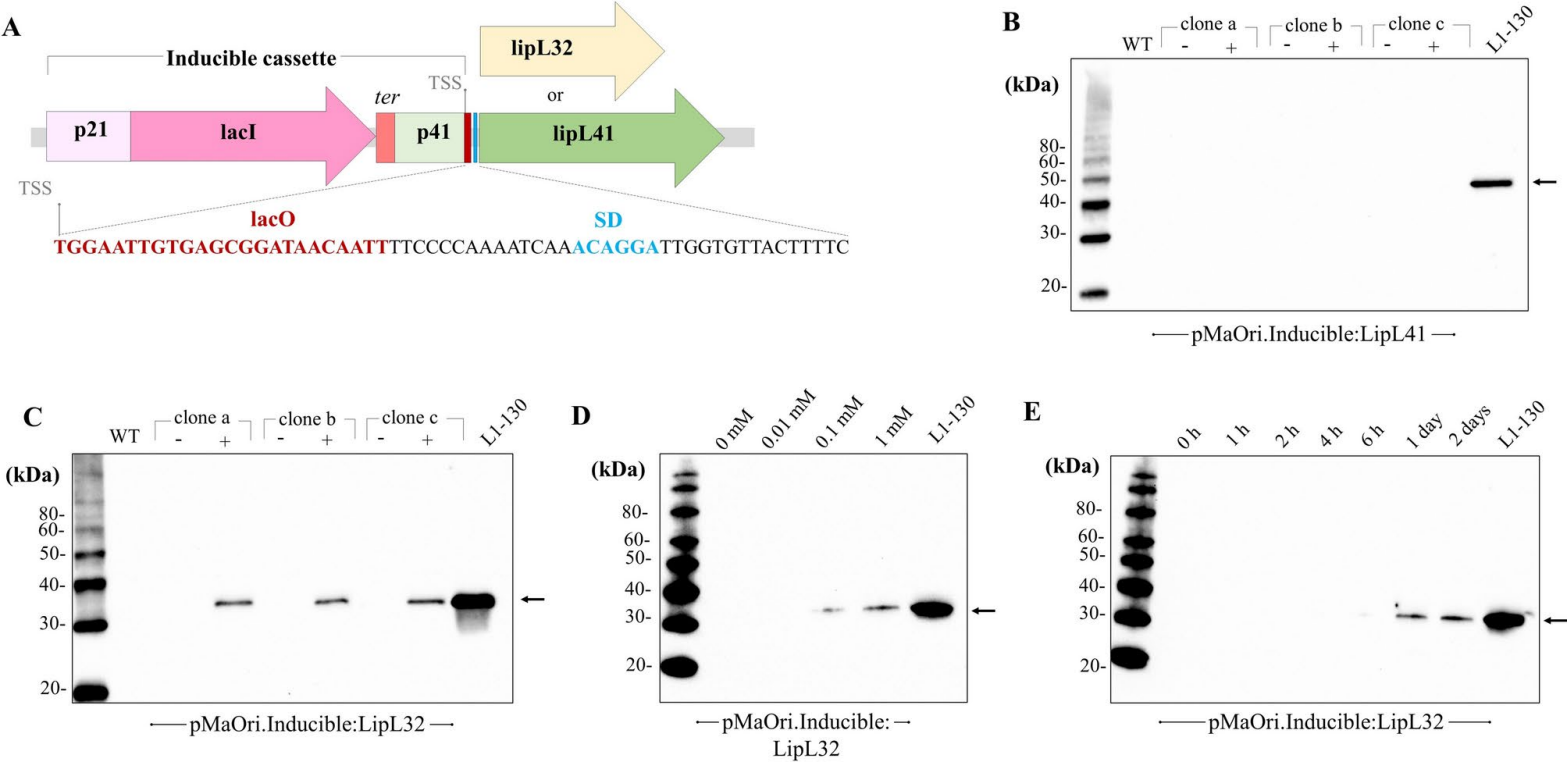
Validation of an IPTG-inducible cassette in ***L. biflexa***.(**A**) An inducible cassette developed for *Leptospira* spp. comprising the *lipL21* promoter directing the transcription of codon optimized lac repressor LacI, followed by a *B. burgdorferi* transcription terminator. The constitutive *lipL41* promoter was used to transcribe the reporter gene (either *lipL41* or *lipL32*), with inclusion of the lacO sequence (red) after TSS. Shine-Dalgarno sequence (SD, blue) is responsible for ribosome binding. *L. biflexa* cells containing the plasmid for IPTG-controlled expression of LipL41 (pMaOri.Inducible:LipL41) (**B**) or LipL32 (pMaOri.Inducible:LipL32) (**C**) were grown in liquid media in the absence (-) or presence ( +) of IPTG and protein expression evaluated by immunoblot. Controlled LipL32 expression was also evaluated in different concentrations of IPTG (**D**) and times of induction (**E**). Cell lysates from *L. interrogans* serovar Copenhageni strain Fiocruz L1-130 were used as a control.

The promoter for the highly expressed gene *lipL21*^39^ from *L. interrogans* serovar Copenhageni strain Fiocruz L1-130 was used to express the codon optimized Lac repressor (LacI), followed by a *Borrelia bmpB *gene transcription terminator^40^. For inducible protein expression, the promoter for the highly expressed gene *lipL41 *was used and included 253 pb upstream from the transcription start site (TSS)^41^ immediately after the TSS, the Lac operator (lacO) sequence was included, followed by the 5’ UTR from *lipL41*, which includes the putative Shine-Delgarno (SD) sequence, and the CDS for *lipL41* **(**Fig. 1A). The entire sequence was synthetized by GeneArt and included the restriction sites *Xba*I and *Not*I at the 5’ and 3’ends, respectively. The cassette was ligated into the pMaOri plasmid^42^, previously digested with the same enzymes, in a T4 ligase-mediated reaction. The final plasmid was named pMaOri.Inducible:LipL41. A plasmid named pMaOri:LipL41, without the IPTG-inducible cassette, was made by cloning the *lipL41* wild-type cassette into the pMaOri backbone by Gibson assembly using amplicons generated by PCR with primers for *lipL41* as listed in **Supplementary Table 1.**

The plasmid pMaOri.Inducible:LipL32 was prepared by substituting the *lipL41* CDS with that of the *lipL32* CDS. The plasmid backbone and inducible cassette, excluding the *lipL41* CDS, were amplified from pMaOri.Inducible:LipL41 using Q5 DNA polymerase (New England Biolabs) with primers pMaOriInducibleF and InducibleCassetteR. The *lipL32* CDS was amplified from genomic DNA of *L. interrogans* serovar Copenhageni strain Fiocruz L1-130 with primers LipL32 F and R. Both fragments were ligated by Gibson Assembly according to manufacturer’s specifications.

### Conjugation of *Leptospira* spp. and transconjugant recovery

The conjugative *E. coli* strain β2163 was transformed by heat-shock with each of the recombinant plasmids described herein. Individual colonies were selected from agar plates, confirmed by colony PCR, and propagated in liquid media. *E. coli* to *Leptospira *spp. conjugation protocols were performed as previously described^18,19^. Transconjugant *Leptospira* were recovered from filter membranes after 24 h conjugation and plated onto EMJH (for saprophytic strains) or HAN (pathogenic strains) solid media containing 40 µg/mL spectinomycin, in the presence or absence of 1 mM IPTG. Plates were incubated at 29 °C until colonies were visible.

### Inclusion of the inducible cassette in pMaOri.dCas9 and pMaOriCas9NHEJ plasmids

The *S. pyogenes* constitutive *cas9 *promoter within pMaOri.dCas9^17^ and pMaOriCas9NHEJsmegmatis^24^ was substituted with the IPTG-inducible cassette by Gibson Assembly. Briefly, PCR amplification of plasmid pMaOri.dCas9 with primers pdCas9 F and R or amplification of plasmid pMaOriCas9NHEJsmegmatis with primer pdCas9 F and pNHEJ R resulted in amplicons without the *S. pyogenes* promoter. The inducible cassette was amplified from plasmid pMaOri.Inducible:LipL41 with primers cassette_dCas9 F and R for ligation into the dCas9 plasmid, or primers cassette_NHEJ F and cassette_dCas9 R for ligation into the Cas9NHEJ plasmid. Appropriate amplicons were then used for Gibson Assembly (New England BioLabs) reactions according to manufacturer’s instructions. Ligated reactions were used to transform *E. coli* β2163 strain as described above and recovered colonies were evaluated by colony PCR to confirm inclusion of the inducible cassette with primers InducibleCassette F and R. The final plasmids pMaOri.Inducible:dCas9 and pMaOriNHEJ.Inducible:Cas9 were confirmed by whole sequencing with Nanopore long-read technology (Plasmidsaurus Inc, Arcadia, California).

### Inclusion of sgRNA cassettes in the inducible plasmids

sgRNA cassettes previously made for *L. biflexa dnaK* (LEPBI_RS16560) and *ompL1* (LEPBI_RS04010), or for *L. interrogans lipL21* (LIC_RS00055), *lipL41* (LIC_RS15260) and *ompL1* (LIC_RS05025) were amplified from plasmids with primers sgRNA F and R, and ligated to *Xma*I digested plasmids pMaOri.Inducible:dCas9 or pMaOriNHEJ.Inducible:Cas9 by Gibson Assembly at a 1(plasmid):10(amplicon) molar ratio. For sgRNA cassettes targeting the non-coding RNase P (LEPBI_RS18770), pMaOri.dCas9sgRNAlipL21 was used as template for PCR amplification using primers RNaseP_sense F and R and RNaseP_antisense F and R to substitute the protospacer within the plasmid for the ones targeting the sense and antisense strands of RNase P, respectively. Amplicons were circularized by Gibson Assembly. Recombinant plasmids were then used for sgRNA amplification, following ligation into *Xma*I digested pMaOri.Inducible:dCas9, as above mentioned.

The conjugative *E. coli* β2163 was transformed with the ligation reactions by heat-shock and cells were seeded onto LB plates containing 0.3 mM DAP and 40 µg/mL spectinomycin. Positive clones containing the recombinant plasmids were validated by PCR with primers Inducible cassette F and R, and pMaOri2 F and R. Targets and selected protospacers are listed in Table 1.

**Table 1:** Target and protospacer used in the sgRNA cassettes.

Target	Protospacer (5′→3’)	Species
*dnaK* (LEPBI_RS16560) coding strand	ACACGAGCCGCAATTTCTTG	*L. biflexa*
*ompL1* (LEPBI_RS04010) coding strand	CGAAACGTACGTTTTCTTTG	*L. biflexa*
RNase P (LEPBI_RS18770) sense strand	TACTTGAGATTGCACATCGG	*L. biflexa*
RNase P (LEPBI_RS18770) antisense strand	CGTTATGAGCCTGTCCCGCC	*L. biflexa*
*ompL1* (LIC_RS05025) coding strand	GCCGCCAGTAGTTCTATCGA	*L. interrogans*
*lipL21* (LIC_RS00055)	AACGTCTTTCGGATCGGATC	*L. interrogans*
*lipL41* (LIC_RS15260)	CTACGTTACGAATGGTTCCG	*L. interrogans*

### Validation of IPTG-inducible protein expression

To validate the IPTG-inducible cassette for controlled protein expression in *Leptospira*, colonies were recovered from agar plates after conjugation and grown in liquid medium without or with IPTG (as indicated) until mid-late log phase (2–5 × 10^8^/mL). Cells were harvested by centrifugation (10,000 × *g*, 15 min), washed twice with PBS and cell lysates prepared by boiling at 95 °C for 10 min in SDS-PAGE loading buffer for gel electrophoresis on either 12% or 4–15% polyacrylamide gels (BioRad). Protein visualization was achieved by staining with Sypro Ruby (Invitrogen, CA) according to the manufacturer’s instructions. For immunoblotting, proteins were electrotransferred onto polyvinylidene difluoride (PVDF) membranes (BioRad) using a semidry transfer method. Membranes were then blocked with SuperBlock (PBS) Blocking Buffer (Thermo) for 1 h, followed by incubation with primary rabbit antibodies (anti-LipL32, anti-LipL21 and anti-LipL41 at 1:4,000, OmpL1 at 1:1,000, anti-Cas9 at 1:2,000 dilution) in blocking buffer for 1 h at room temperature. After three washes with PBS containing 0.1% Tween 20 (PBS-T), membranes were incubated with horseradish peroxidase-conjugated secondary antibodies (1:4,000 dilution) in blocking buffer for 1 h at room temperature. Following six washes, antigen reactivity was detected using Clarity Max ECL (BioRad), and luminescence was captured with a ChemiDoc MP Imaging System (BioRad).

### Validation of IPTG-induced dCas9 function to silence gene expression

*E. coli* β2163 containing the plasmids for IPTG-induced dCas9 expression and with sgRNA targeting the putative leptospiral genes *dnaK* (LEPBI_RS16560)*, ompL1* (LEPBI_RS04010) or the non-coding RNaseP (LEPBI_RS18770) were used for conjugation with wild-type *L. biflexa*. After recovery of transconjugants from filters, suspensions were spread onto EMJH plates containing 40 µg/mL spectinomycin, with or without 1 mM IPTG. The presence of IPTG induces expression of dCas9 and thus the subsequent silencing of genes targeted by the sgRNA acts as a first indication as to whether a targeted gene provides an essential function as evidenced by no, or a drastically reduced number of, recovered colonies compared to plating in the absence of IPTG. To further validate the essentiality of the gene of interest, individual colonies were then selected from plates without IPTG and inoculated (10^5^/mL) into liquid medium plus spectinomycin, with or without IPTG. To confirm that the addition of IPTG to plates was not impairing colony formation, a control containing pMaOri.Inducible:dCas9 with no sgRNA was included in each experiment, and cells were plated in the presence or absence of IPTG. When growth was observed in the presence of IPTG, cells were evaluated for expression of dCas9. IPTG-induced gene silencing by dCas9 targeting *ompL1* and *lipL41* was also performed in pathogenic *L. interrogans,* using conjugation methods for pathogenic species as described above.

### Recovery of knockout mutants by IPTG-induced Cas9 expression in conjunction with constitutively expressed NHEJ machinery from *M. smegmatis*.

The plasmid pMaOriNHEJ.Inducible:Cas9 was designed for IPTG-inducible expression of Cas9 nuclease and constitutive expression of two *M. smegmatis* proteins, LigD and Ku, responsible for DSB repair after Cas9 DNA target cleavage. This plasmid, with or without a sgRNA targeting *lipL21*, was delivered by conjugation to pathogenic *Leptospira*. Recovered cells were plated onto HAN media containing 40 µg/mL spectinomycin, with or without 1 mM IPTG. Plates were incubated at 29 °C until colonies were visible, and colonies were selected from plates without IPTG for growth in liquid media, with or without IPTG. Cultures were monitored daily to account for any delayed growth in the presence of IPTG. Cell lysates were evaluated by immunoblotting, and upon confirmation of knockout phenotypes, mutant cultures were diluted to 10^3^ cells/mL and plated onto HAN plates for individual colony isolation. Individual colonies were selected, grown in liquid media for extraction of DNA, PCR amplification of mutated sites, and amplicon sequencing. The *lipL21* gene was amplified by PCR with primers spanning approximately 200 bp from the Cas9 cutting site **(**Table 1**)**. PCR products were then cleaned up with ExoSAP-IT (Applied Biosystems, Foster City, California, USA) and labeled using the Big Dye Terminator v3.1 cycle sequencing reagent (Applied Biosystems). Sequencing was performed using the ABI 3130XL Genetic Analyzer.

## Results

### Validation of IPTG-induced expression in the surrogate *L. biflexa*

The *lipL41* gene was initially selected to validate expression of the IPTG-inducible cassette in *L. biflexa*. For that, the *lipL41* CDS, from start codon ATG to 128 nucleotides downstream of the stop codon, was utilized. The expression of codon optimized lac repressor, LacI, is driven by the constitutive and strong *lipL21* promoter. To ensure proper termination, the *B. burgdorferi bmpB* gene intrinsic terminator downstream of the *lacI* coding sequence was included **(**Fig. 1A**)**. Next, the *lipL41* promoter spanning 254 nucleotides from the experimentally defined transcription start site (TSS), as reported by Zhukova et al. in (2017), was used, followed by the lacO sequence and the *lipL41* 5’UTR, including the putative Shine-Dalgarno sequence **(**Fig. 1A**)**.

Plasmid pMaOri containing the inducible cassette for *lip41* expression was delivered to *L. biflexa* by conjugation with *E. coli* cells, and recovered colonies grown in liquid media to generate whole-cell lysates for immunoblotting. No LipL41 protein could be detected in the recombinant *L. biflexa* cells, indicating that this protein is not expressed in the saprophytic strain **(**Fig. 1B**)**. As an alternative method to demonstrate IPTG-controlled expression, the *lip41* CDS was substituted by the *lipL32* CDS **(**Fig. 1A**)**, since LipL32 protein has been successfully expressed in *L. biflexa *previously^17^. Colonies were randomly selected from plates and grown in liquid media with or without IPTG. Subsequent analysis by immunoblotting demonstrated that expression of LipL32 was detected only in the presence of the inducer, indicating controlled gene expression by the inducible cassette (Fig. 1C). No LipL32 was detected in the absence of IPTG even after overexposure of immunoblots (data not shown). *L. biflexa* cells containing the inducible cassette for *lipL32* were used to determine an optimal concentration of 1 mM IPTG for expression of the inducible cassette **(**Fig. 1D**)**. LipL32 was faintly detected at 6 h post IPTG induction, with peak expression apparent after 1 day **(**Fig. 1E**)**.

### IPTG-induced expression of dCas9 and Cas9 proteins in *L. biflexa*

**Fig. 2:**
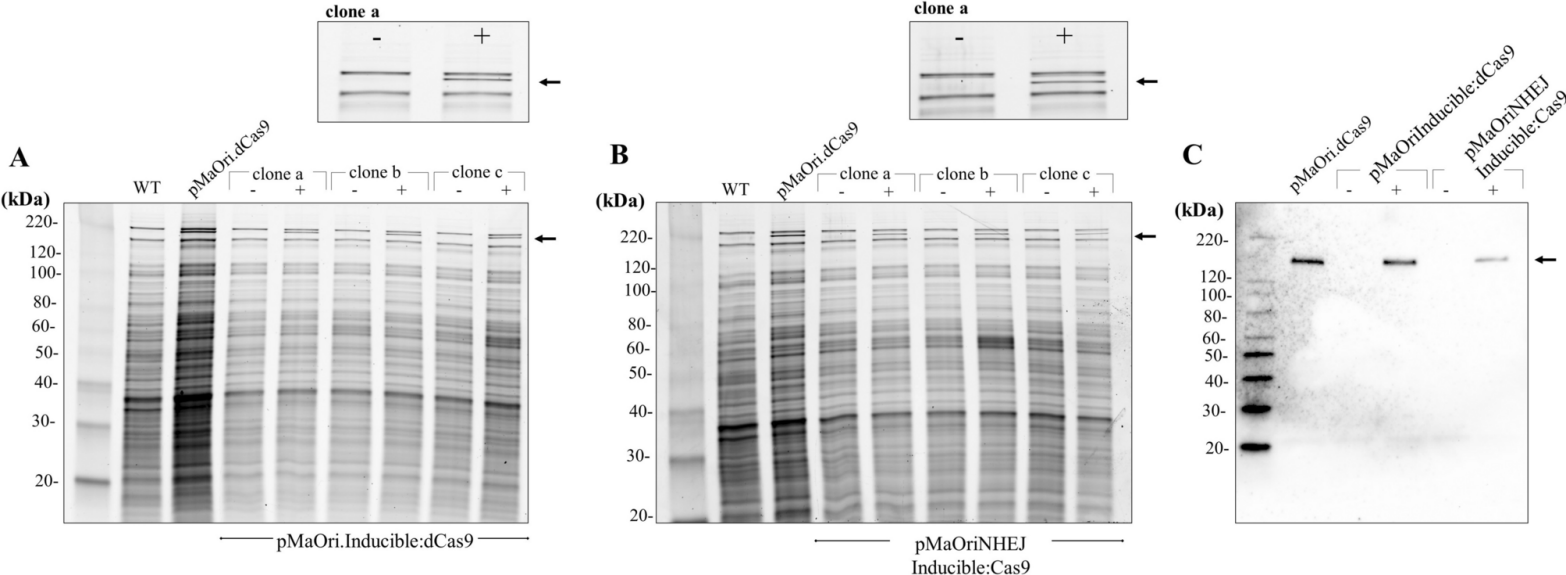
Evaluation of IPTG-induced expression of dCas9 and Cas9 in ***L. biflexa*.** Total protein from *L. biflexa* cells containing the plasmids pMaOri.Inducible:dCas9 (**A**) and pMaOriNHEJ.Inducible:Cas9 (**B**) and grown without (-) or with ( +) IPTG were evaluated by Sypro Ruby staining. Upper panel highlights the appearance of dCas9 or Cas9 protein in the presence of IPTG. Wild-type (WT) and *L. biflexa* containing the plasmid pMaOri.dCas9 were used as controls. dCas9 and Cas9 controlled expression was validated by immunoblotting with anti-Cas9 antibodies (**C**).

The native *S. pyogenes* promoter within the pMaOri.dCas9 and pMaOri.Cas9NHEJsmegmatis was substituted for the IPTG-inducible cassette by PCR, resulting in the generation of plasmids pMaOri.Inducible:dCas9 and pMaOriNHEJ.Inducible:Cas9, respectively. Final plasmids were confirmed by sequencing and their genetic elements and complete sequence are presented in **Supplementary Fig. 1** and **Supplementary Table 2,** respectively. Recombinant *L. biflexa* colonies were grown in media with or without IPTG for validation by SDS-PAGE and immunoblotting with anti-Cas9 antibodies. An ~ 160 kDa band was detected in whole cell lysates of cells containing the control plasmid pMaOri.dCas9, in which dCas9 is constitutively expressed, as well as in the clones containing the inducible cassette for dCas9 in the presence of IPTG **(**Fig. 2A** and upper panel)**. The same profile was observed in the cells containing the inducible cassette for Cas9 **(**Fig. 2B** and upper panel)**. Clones containing each construct were evaluated by immunoblotting, corroborating the IPTG-induced expression of dCas9 and Cas9, with no bands being detected in the absence of the inducer. These encouraging results prompted further experiments to target genes considered essential for *Leptospira* by using IPTG-induced gene silencing with dCas9, and IPTG-induced gene knockout and recovery with Cas9 expression in conjunction with NHEJ proteins.

### IPTG-induced gene silencing to identify essential genes

**Fig. 3:**
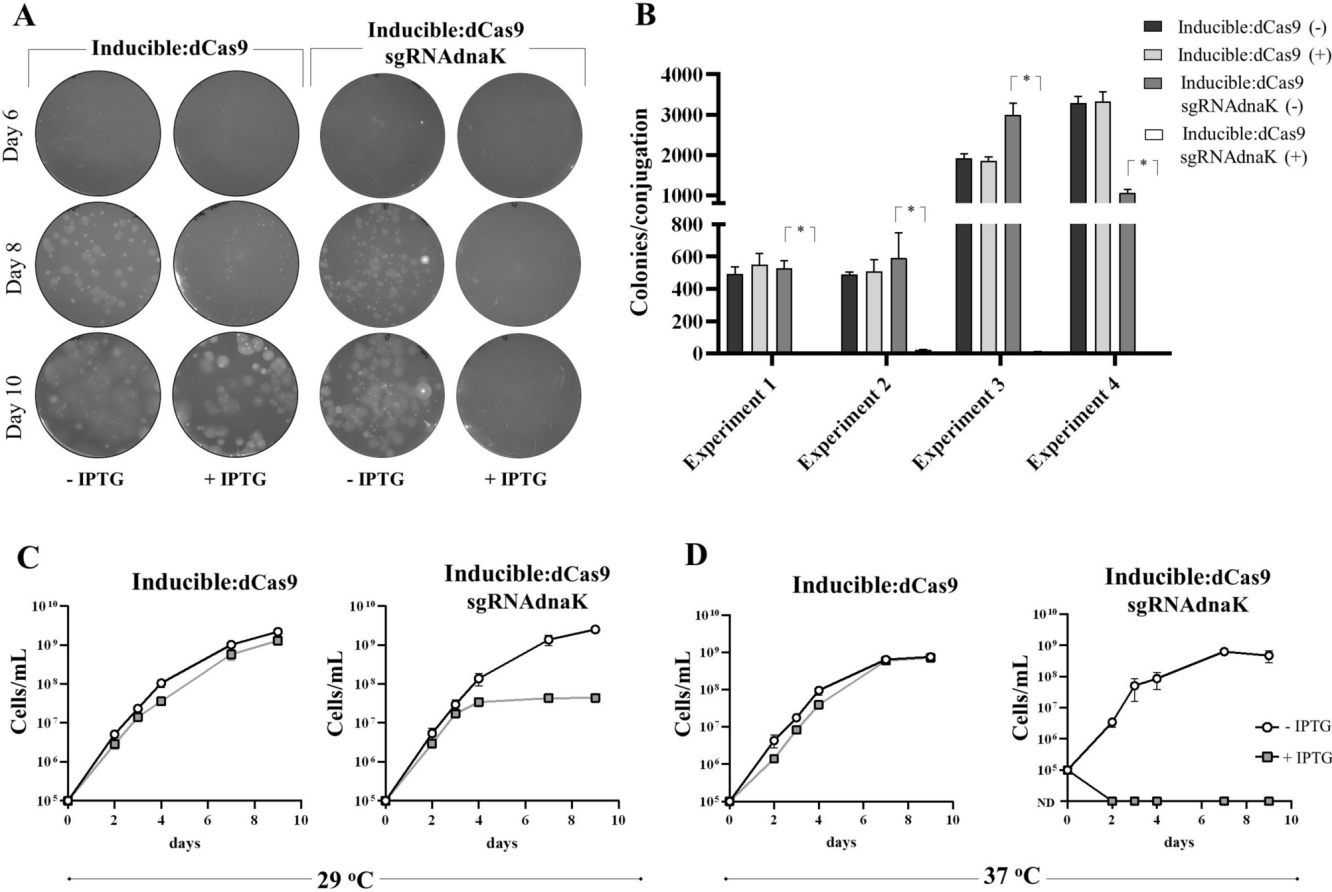
Evaluation of conditional ***dnaK*** knockdown mutants in***L. biflexa*. **(**A**) *L. biflexa* cells containing the plasmids pMaOri.Inducible dCas9 alone or with a sgRNA targeting the *dnaK* gene were seeded onto EMJH media containing spectinomycin without (-) or with ( +) 1 mM IPTG. Colony formation was monitored and recorded at different time points. (**B**) Colonies observed across four independent experiments. Number of colonies recovered in the absence or presence of IPTG was compared by t-test (*p < 0.05). Colonies from plates without IPTG (n = 3) were picked, cells released from the agar and inoculated in liquid HAN media containing spectinomycin at 10^5^ cells/mL, in the absence or presence of IPTG, and growth was monitored daily at both 29 (**C**) and 37 °C (**D**).

Cassettes containing sgRNA targeting expression of the *dnaK* gene in *L. biflexa* were obtained by PCR and ligated into pMaOri.Inducible:dCas9. After conjugation, cell mixtures were plated onto EMJH plates containing spectinomycin, with or without 1 mM IPTG. After 6 days of incubation, colonies began to be apparent in all groups in the absence of IPTG. For those cells containing the control plasmid with no sgRNA cassette, the presence of IPTG, and therefore the expression of dCas9, delayed colony formation, since colonies were apparent at 8 days (Fig. 3A, one representative experiment out of four). When sgRNA targeting *dnak* was included in the plasmid, the presence of IPTG abrogated or drastically reduced colony formation, indicating that the absence of the chaperone DnaK impaired leptospiral growth on plates **(**Fig. 3A and B**)**. Figure 3B depicts the number of colonies recovered after each conjugation experiment, in both the absence and presence of IPTG. These results agree with previous attempts to recover *dnak *knockdown mutants by CRISPRi^17^.

To further confirm the essential role of the DnaK protein on leptospiral physiology, individual colonies were selected from plates without IPTG, and cells inoculated into liquid HAN media, which supports the growth of leptospires at both 29 and 37 °C, supplemented with spectinomycin with or without IPTG. At 29 °C, cells containing plasmid with no sgRNA displayed similar growth curves in the presence or absence of IPTG **(**Fig. 3C**)**; however, in the presence of IPTG, cells containing pMOri.Inducible:dCas9sgRNAdnaK displayed abnormal morphology, and an early stationary phase with lower cell densities (~ 10^7^/mL) **(**Fig. 3C, **Supplementary Fig. 1)**.

Incubation of cells with IPTG at 37 °C completely abolished growth in the conditional *dnaK* knockdown mutant, indicating that the chaperone DnaK is essential for cells to cope with higher temperatures **(**Fig. 3D**)**. Even though leptospires with an unusual morphology were observed at day 1 **(Supplementary Fig. 2)**, no cells could be detected beyond day 2 in the presence of IPTG, and in contrast to growth in the absence of IPTG, or control cells with no sgRNA **(**Fig. 3D**)**.

### IPTG-inducible gene silencing of non-coding RNA

To further corroborate the applicability of IPTG-inducible gene silencing to establish whether targets are essential, and to demonstrate the feasibility of silencing non-coding RNA in *Leptospira*, a sgRNA cassette targeting RNAseP was constructed. Both IPTG-inducible and constitutively expressed dCas9 strategies were tested and included a sgRNA targeting the antisense strand of RNAseP for partial silencing^43^^,^^17^.

**Fig. 4:**
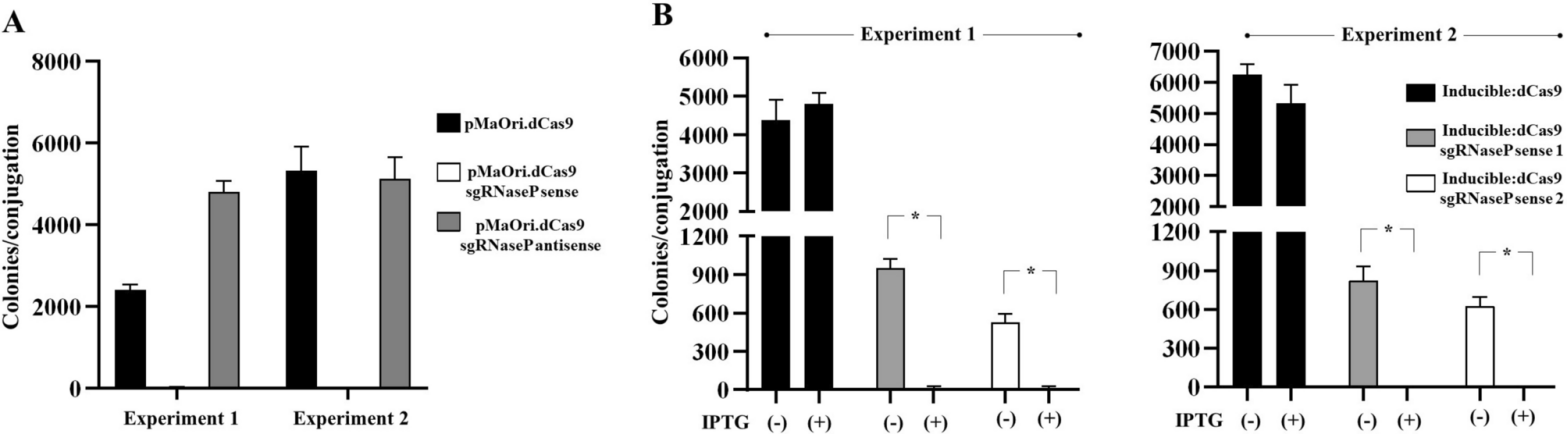
Silencing of the ncRNA RNase P in ***L. biflexa***. (**A**) *L. biflexa* cells containing plasmids pMaOri.dCas9 alone, or with sgRNA cassettes for pairing to the sense (complete silencing) or antisense (incomplete) strand of the RNaseP sequence, were plated onto EMJH media containing spectinomycin. Colonies obtained from two independent experiments were counted. (**B**)Validation of essentiality of ncRNA RNaseP was demonstrated by inducible silencing. Cells harboring the pMaOri.Inducible:dCas9 alone or with the sgRNasePsense were seeded onto EMJH plates with spectinomycin without (-) or with ( +) IPTG. Two independent experiments were performed and at each, two clones of conjugative *E. coli* were used. Number of colonies recovered in the absence or presence of IPTG was compared by t-test (*p < 0.05).

When using the plasmids for constitutive expression of dCas9, colonies could be recovered when cells contained the pMaOri.dCas9 plasmid alone or with the sgRNA targeting the antisense strand of RNAseP. In contrast, a reduced number or no colonies were obtained using the sgRNA targeting the sense strand for RNAseP **(**Fig. 4A**)**. Since reduced colony recovery could also be due to reduced conjugation efficiencies, the IPTG-inducible plasmid was used for complete silencing of RNAseP. Plating of transconjugants onto EMJH plates with or without IPTG determined that indeed, silencing expression of RNAseP affects cell viability since colony formation was drastically impaired in the presence of IPTG **(**Fig. 4B**)**. Two clones of *E*. *coli* β2163 containing the pMaOri.Inducible:dCas9sgRNasePsense were tested. Results corroborate that inducible silencing is a feasible strategy to validate the essential requirement of genes as well as non-coding RNA, and represents the first demonstration of silencing of this class of macromolecules in *Leptospira* spp.

### Reassessing the essential role for the OmpL1 porin in *Leptospira* spp.

**Fig. 5:**
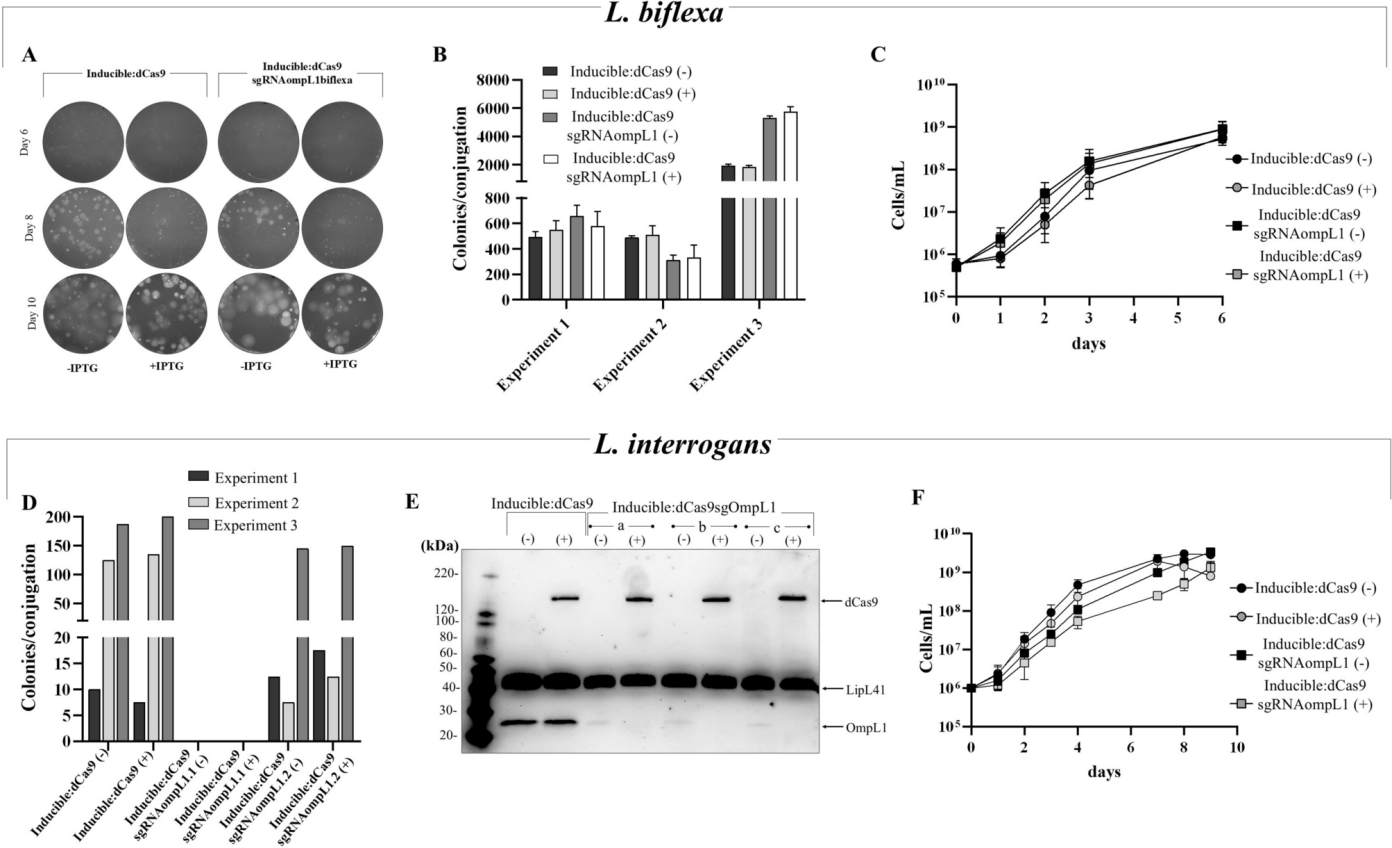
OmpL1 porin silencing in ***L. biflexa* and *L. interrogans*. **(**A**) *L. biflexa* cells containing plasmid pMaOri.Inducible:dCas9 alone, or with a sgRNA cassette targeting the *ompL1* gene, were seeded onto EMJH media containing spectinomycin without (-) or with ( +) 1 mM IPTG. Colony formation was monitored and recorded at different time points. (**B**) Three independent experiments were performed, and numbers of colonies recovered in the absence or presence of IPTG were recorded. (**C**) Cell viability was also demonstrated in liquid media in the absence (-) or presence ( +) of IPTG. (**D-F**) Silencing was also performed in the ompL1 ortholog in *L. interrogans* strain Fiocruz L1-130. (**D**) Three independent conjugation experiments were performed and for each, two clones of conjugative *E. coli* containing the plasmid for *ompL1* silencing were used. (**E**) Colonies from plates without IPTG (n = 2) were picked, cells released from the agar and inoculated in liquid HAN media containing spectinomycin in the absence (-) or presence ( +) of IPTG. Cells at mid-log phase were recovered and cell lysates evaluated by immunoblotting. (**F**) Viability was also confirmed by growth curves in liquid EMJH containing spectinomycin, in the presence or absence of IPTG.

A sgRNA cassette targeting the *ompL1* gene of *L*. *biflexa* was generated and the final plasmid was delivered to leptospiral cells by conjugation. Cells were plated onto EMJH media containing spectinomycin without (-) or with ( +) IPTG, and colony growth was monitored daily. As seen in Fig. 5A, colonies were observed in both control (no sgRNA) and gene knockdown groups (pMaOri.Inducible:dCas9sgRNAompL1biflexa), in either the absence or presence of IPTG, suggesting that OmpL1 silencing is tolerated by *L*. *biflexa*. Colonies were enumerated and results of three independent experiments are presented in Fig. 5B. Results corroborate that cells remain viable in the absence of OmpL1 porin, and as confirmed by cell growth in liquid media **(**Fig. 5C**)**. Since antibodies specifically targeting OmpL1 in the saprophyte *L. biflexa* are unavailable, gene silencing of *ompL1* in the pathogen *L. interrogans* serovar Copenhageni strain Fiocruz L1-130 was performed, not only to demonstrate silencing by probing the target protein with specific antibodies, but also to confirm that *L. interrogans* cells were viable in the absence of the porin. Colonies of *L*. *interrogans* could be recovered after conjugation from HAN plates without or with IPTG across 3 independent experiments **(**Fig. 5D**)**.

Colonies were recovered from agar plates without IPTG and propagated in liquid media containing spectinomycin without (-) or with ( +) IPTG. Mid-log phase leptospires were collected and whole-cell lysates were evaluated by immunoblotting with anti-OmpL1 and anti-Cas9 antibodies **(**Fig. 5E**)**. As expected, dCas9 was detected only in cells grown in the presence of IPTG; no dCas9 expression was observed in the absence of IPTG even after overexposure of membranes (not shown). However, diminished expression of OmpL1 was observed in transconjugants with the plasmid pMaOri.Inducible:dCas9sgRNAompL1interrogans in the absence of IPTG when compared with those without the sgRNA for *ompL1*, suggesting that basal “leaky”, yet undetectable, levels of dCas9 are present. Nevertheless, the presence of IPTG completely abolished expression of OmpL1, as confirmed by immunoblot **(**Fig. 5E**)**. Nor was expression of OmpL1 required for growth in liquid media **(**Fig. 5F**)**. “Leaky” silencing was also observed when *L. interrogans* cells contained the plasmid pMaOri.Inducible:dCas9sgLipL41 targeting expression of LipL41 **(Supplementary Fig. 3)**.

### Recovery of knockout mutants for *lipL21 *in pathogenic *Leptospira*

**Fig. 6:**
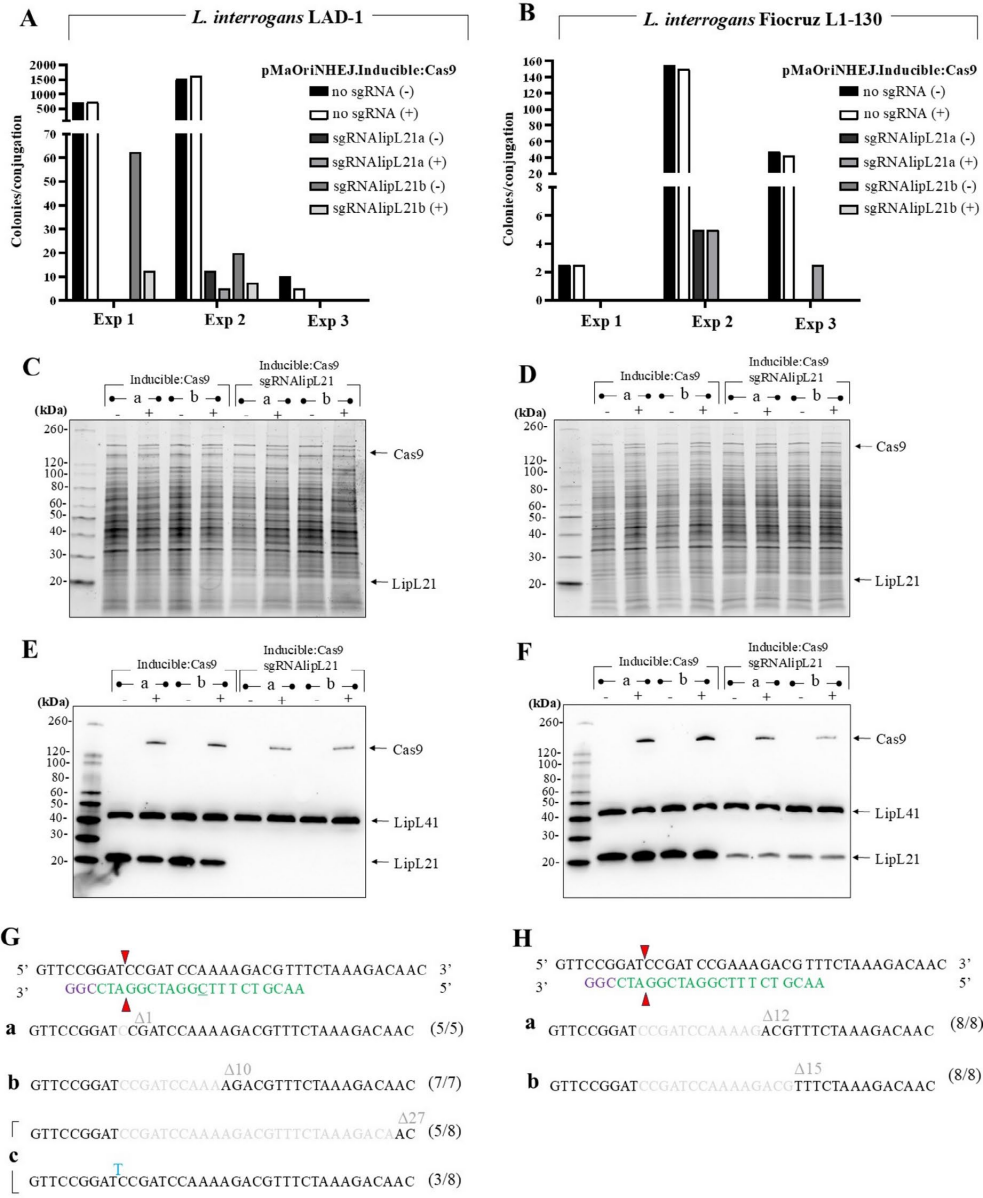
***lipL21*** knockout by inducible Cas9 in different strains of ***L. interrogans*.** Conjugation reactions for *L. interrogans* serogroup Canicola strains LAD-1 (**A**) and serovar Copenhageni strain Fiocruz L1-130 (**B**) were seeded onto HAN plates with spectinomycin, without (-) or with ( +) 1 mM IPTG. Numbers of recovered colonies after 3 independent experiments were recorded, utilizing two clones of *E. coli* with the plasmid containing the sgRNA targeting *lipL21* in each experiment. Control plasmid with no sgRNA, therefore no DSB, was also employed. Distinct colonies from the plates without IPTG were selected, grown in liquid media without or with IPTG, and whole cell lysates were by SDS-PAGE followed by Sypro Ruby staining (**C**, LAD-1; **D**, L1-130) and immunoblotting (**E**, LAD-1; **F**, L1-130). Cultures (a-c for LAD-1; a and b for L1-130) were plated for individual colonies, which were selected and *lipL21* mutations were assessed by sequencing. (**G**) Mutation in distinct colonies (mutation/total colonies) from original colonies “a-c” in LAD-1, and (**H**) from original colonies “a and b” in L1-130. Sequence in green denotes the protospacer contained in the sgRNA, including a mismatch (underlined) for LAD-1, followed by the PAM (purple, not contained in the sgRNA sequence). Cas9 cleavage sites are indicated by the red triangles.

A sgRNA cassette targeting the *lipL21* gene of *L. interrogans* strain Fiocruz L1-130 was included into the pMaOriNHEJ.Inducible:Cas9 plasmid. Conjugation reactions for *L. interrogans* serogroup Canicola strain LAD-1 or serovar Copenhageni strain Fiocruz L1-130 were plated in HAN media without (-) or with ( +) IPTG, and colonies were enumerated **(**Fig. 6A and B**)**. For strain LAD-1, which displayed overall higher conjugation efficiencies than strain L1-130, the absence of IPTG favored colony recovery, despite the variability in conjugation efficiencies observed across three independent experiments. Nevertheless, a reduced number of colonies were retrieved in groups containing the sgRNA compared to those without (pMaOriNHEJ.Inducible:Cas9), even in the absence of IPTG, implying that to some extent, lethal DSB were occurring due to a basal “leaky” protein expression of Cas9, and as observed for the IPTG-induced dCas9 cassette.

Colonies from agar plates without IPTG were selected, grown in liquid HAN without or with IPTG and whole cell lysates prepared for evaluation of total protein profiles **(**Fig. 6C and D**)** and immunoblotting **(**Fig. 6E and F**)**. For both strains LAD-1 and L1-130, Cas9 expression could be readily observed in total protein profiles only in the presence of IPTG, with no detectable expression of LipL21 in proteins from cells grown in the absence or presence of IPTG. Immunoblotting confirmed the absence of LipL21 protein for LAD-1 cells, even without IPTG, confirming a basal, yet undetectable, expression of Cas9; for L1-130 cells, reduced levels of LipL21 were observed, suggesting that the mutation inflicted into *lipL21* gene affected protein expression.

Populations derived from each selected colony (“a-c” for strain LAD-1; “a” and “b” for strain L1-130) were plated and individual colonies were selected to confirm mutation by gene sequencing **(**Fig. 6G**)**. For strain LAD-1, all colonies from population “a” and “b” presented with a 1 and 10 nucleotide deletion, respectively, starting from the Cas9 cleavage site. For colony “c”, and out of 8 clonal isolates evaluated, 5 presented with a deletion of 27 nucleotides, and 3 presented with a 1 nucleotide insertion; this suggests that cells underwent DSB after at least one generation, resulting in a mixed population of mutant cells within the same colony. Since the sgRNA used in this study targets the 3’ end of *lipL21*, it was unexpected that the slight truncation of the final protein product would so drastically affect protein expression. Of note, the sgRNA for *lipL21* was designed based on the genome of strain L1-130 and is mismatched with the target sequence of strain LAD-1 (underlined nucleotide in the green sequence). The full-length LipL21 protein, excluding the signal peptide, has a predicted molecular mass of 17.82 kDa. The predicted molecular mass for LipL21 is 15.26 kDa in clone “a” (containing a 1 nucleotide deletion), 13.53 kDa in clone “b” (containing 10 nucleotides deletion), and either 16.90 kDa (27 nucleotides deletion, in frame mutation) or 14.51 kDa (1 nucleotide insertion) for clone “c”.

Analysis of mutations for *lipL21* in colonies of strain L1-130 showed that all those derived from clone “a” displayed a 12-nucleotide deletion (resulting in a protein of 17.45 kDa) and those derived from clone “b” had a 15-nucleotide deletion (resulting in a truncated LipL21 of 17.33 kDa) **(**Fig. 6H**)**. Despite this minor reduction on the protein’s molecular mass, it was again observed that protein expression was affected.

## Discussion

The negative regulation of the lactose operon in *E. coli *is achieved by a DNA binding repressor called LacI^44–46^. The lac repressor acts as a homotetramer, consisting of two functional homodimers of LacI polypeptides^44,46^, capable of binding to a DNA sequence termed the lac operator (lacO), located upstream of the structural genes of the lac operon. When bound to allolactose, or its analog IPTG, LacI undergoes allosteric modification which in turn changes its ability to bind DNA, derepressing transcription of the operon. The regulatory elements of the lac operon have been extensively applied to molecular and system biology, including the spirochete *Borrelia burgdorferi*^47^^,^^48^.

It has been previously shown that the lac system is functional in *Leptospira,* being used to express heterologous *gfp*^49^*.* In this early version of the IPTG-inducible cassette, the *B. burgdorferi* promoter *flaB* was used to transcribe the *lacI* gene, codon optimized for *Borrelia*, followed by the *hsp10* promoter and lacO sequence. These results indicated that *Leptospira *cells are permeable to IPTG, most probably via passive diffusion^50^, since *Leptospira *spp. lack a lactose permease^51^. These results prompted us to develop an optimized inducible cassette, comprising promoters with high transcriptional activity and a *lacI* sequence codon optimized for *Leptospira* spp.

When LipL41 protein was used to validated conditional protein expression in the surrogate *L. biflexa*, no protein band corresponding to the heterologous protein could be observed; we further used the wild type *lipL41* cassette from *L. interrogans*, without any exogenous genetic elements, and recombinant *L. biflexa* cells were still incapable of expressing LipL41. This is probably due to the absence of the *lep*coding sequence in the constructs, since Lep is a molecular chaperone essential for the stable expression of LipL41^52^. Nevertheless, inclusion of *lipL32 *CDS to the IPTG-inducible cassette validated the successful protein expression only in the presence of the inducer, whereas no band was observed in its absence, suggesting a well-regulated system. However, it is well established that transcription from the lac promoter is never completely abolished, and some basal “leaky” levels of mRNA can be expected^53^.

When the IPTG-inducible cassette was used for controlled dCas9 expression, the “leaky” basal, yet undetectable, expression of dCas9 was indirectly demonstrated. Even though no dCas9 protein could be detected when *L. biflexa* and *L. interrogans* cells grew in the absence of IPTG, a reduced expression of the target proteins was observed when *L. interrogans* contained plasmids for IPTG-induced expression of dCas9 and sgRNA targeting either OmpL1 or LipL41, compared to the control cells harboring the plasmids with no sgRNA, even in the absence of the inducer. However, the addition of IPTG and consequently observable dCas9 expression resulted in complete silencing of the target genes. Basal expression of dCas9 was also observed in the IPTG-inducible system for *B. burgdorferi*^47^, more pronouncedly when the strains carried the system in a shuttle vector which displays a 5:1 ratio to the chromosome. Leakage was also observed in the previously constructed IPTG-inducible cassette for *L. biflexa*^49^.

Interestingly, even with target genes affected by basal dCas9 expression, our system could be successfully used to validate essential genes in the model *L. biflexa,*with no deleterious phenotypes being observed in the absence of IPTG. It is worth mentioning that the selected targets for silencing in this work code for highly abundant products, since DnaK is expressed in over 3,000 copies/cell^39^ and RNase P is a major ncRNA in *Leptospira*^41,54^. Nonetheless, when working with less abundant targets for essentiality assessments, the basal leakage of dCas9 and its effect target silencing in the absence of IPTG could be more impactful to the phenotype. Accordingly, when Takacs et al.^47^ used an all-in-one shuttle vector for controlled silencing for *rodA*, *mreB* or *ftsI*, they observed phenotypes consistent with significant basal CRISPRi activity, where they were unable to generate clones containing the plasmid targeting *mreB,* even in the absence of IPTG. The authors also observed a delayed colony formation for *rodA*plasmid-containing transformants, with cells often displaying a phenotype in line with a RodA depletion phenotype, even without IPTG. The lack of observable phenotype in the absence of IPTG in our study could also be related to the 1:1 ratio of pMaOri plasmid, the backbone of our all-in-one plasmid for IPTG-inducible silencing, regarding the leptospiral chromosome^18,19^.

In several bacteria, DnaK is essential for cell growth not only at elevated temperatures but also under optimal conditions^55^. When CRISPRi with constitutive expression of dCas9 was used, no *L. biflexa* colonies could be recovered when the *dnaK *gene was targeted for silencing^17^. IPTG-inducible CRISPRi was used to re-validate these findings, since this novel tool can discern between a failed conjugation or a lethal phenotype by comparison between colony recovery with and without IPTG. Accordingly, agar plates after *L. biflexa* conjugation revealed that the presence of IPTG, and therefore *dnaK* gene silencing, completely annulled colony formation, whereas plates without IPTG displayed similar colony growth rates in comparison to the control group with no sgRNA. Of note, the ability to plate the same conjugation reaction into plates with and without IPTG provides a valuable and more definitive assessment of essential genes, ruling out unsuccessful conjugation experiments.

Evaluation of conditional *dnak* mutants in liquid media revealed that knockdown mutants for the DnaK chaperone are not viable at higher temperatures (37 °C for *Leptospira*), and in agreement with previous observations for *E. coli *at 42 °C^56^ also, and consistent with the slower growth of null *dnaK* mutants in *E. coli* at 30 and 37 °C, *L. biflexa* mutants reached lower cell densities (~ 100-fold) and presented with an abnormal morphology, compared to cells without IPTG. The mutant growth in liquid medium at 29 °C could also be due to some residual DnaK concentrations in cells that were gradually “titrated” after silencing as cells divided, until it reached critical levels interrupting cell growth. Of note, *E. coli *mutants that could grow in liquid but not form colonies in solid media were identified in a library of temperature-sensitive mutants^57^.

Deep RNA sequencing has been used for transcriptome analysis and regulatory RNA discovery in several pathogenic bacteria^41,58–60^. So far, little is known about ncRNA function in *Leptospira* spp. and whether they have an impact upon virulence. To verify the feasibility of the application of CRISPRi to ncRNA silencing in *Leptospira*, the non-coding RNase P target was selected, based on its high expression profile^41^ and its ubiquitous and essential role, since RNase P is involved in the processing of multiple RNA substrates, including tRNAs and small non-coding RNAs^61^. Inactivation of RNase P leads to accumulation of 5′- extended tRNAs and nonviability in *E. coli*^62^^,^^63^.

Using a constitutive CRISPRi strategy, it was shown that the complete absence of RNase P is not tolerated by *Leptospira* cells, even though colony formation was observed when partial silencing was performed. To confirm that indeed the absence of colonies was due to silencing lethality rather than a failed conjugation, the IPTG-induced dCas9 system was used, in conjunction with the sgRNA for complete silencing. Accordingly, while colonies could be recovered in the absence of IPTG, the presence of the inducer completely abolished colony formation, confirming the essential role of RNase P to *Leptospira* and further demonstrating the feasibility of CRISPRi for silencing ncRNAs in these bacteria.

Because of the previously published inability to recover colonies after silencing of *OmpL1*, this porin was proposed as essential for leptospiral biology^35^. Now, taking advantage of the IPTG-inducible silencing, we revisited the role of OmpL1. When both *L. biflexa* and *L. interrogans* cells harbored plasmids for IPTG-inducible dCas9 and sgRNA targeting their respective *ompL1* genes, colonies could be equally recovered in plates with or without IPTG, and conditional mutants displayed similar growth rates in liquid media. Target protein absence and dCas9 conditional expression was demonstrated by immunoblotting in pathogenic *L. interrogans, *corroborating that the recovered cells in liquid media in the presence of IPTG were truly knockdown mutants, expressing dCas9, instead of spontaneous spectinomycin-resistant mutants. In this sense, the lack of transconjugant recovery in our previous publication^35^ could have been due to failed conjugations, consolidating the importance of using the IPTG-inducible CRISPRi.

The concurrent expression of RNA-guided Cas9 and *M. smegmatis* proteins LigD and Ku, which are responsible for the ligation of DSBs, has allowed the recovery of knockout mutants in *Leptospira *spp., with the possibility of plasmid elimination after establishment of mutations^24^. Since both conjugation and DNA repair efficiencies are interdependent events, mutant recovery is somewhat low in this system. To address this gap, a repressed Cas9 expression would allow transconjugant recovery in a first step that is limited only by conjugation efficiencies. This is followed by propagation of cells and Cas9 expression by IPTG and now making mutant recovery dependent on DNA repair frequency. But because of basal leakage of Cas9, DSB and therefore knockout mutagenesis occurred even in the absence of IPTG, but with an apparent better efficiency compared to when conjugation reactions were plated in the presence of the inducer. In addition, strain LAD-1 displayed overall higher conjugation efficiencies. Since a *lipL21* mutant colony for LAD-1 displayed a dual population, it suggests that, since Cas9 is expressed in minor basal concentrations in the cytoplasm of these cells, DSB could be happening after at least one cell replication cycle, also giving time for LigD and Ku proteins to accumulate and more readily and efficiently deal with DNA repair.

In conclusion, we show that the IPTG-controlled expression of heterologous proteins in *Leptospira* spp. and its application for dCas9 and Cas9 expression, further advances the potential for genetic manipulation in these bacteria. The regulated expression of dCas9 enables conditional silencing of target genes and validation of essential genes and non-coding RNAs, providing valuable tools for deeper insights into leptospiral biology and virulence mechanisms. Additionally, the improved recovery of knockout mutants through the controlled Cas9 system, combined with constitutive NHEJ expression, was achieved even in the absence of IPTG due to basal Cas9 expression. This approach facilitates the generation of mutant strains that hold promise for studying virulence phenotypes and enhancing bacterin vaccines for veterinary applications.

## Supplementary Information

Supplementary Information 1.

Supplementary Information 2.

## Data Availability

The original contributions presented in the study are included in the article/Supplementary material. Further inquiries can be directed to the corresponding author.
