# Supplementary material for: Induced protein expression in Leptospira spp. and its application to CRISPR/Cas9 mutant generation
Source: Sci Rep. 2025 Feb 5;15:4334. doi: 10.1038/s41598-025-88633-w (PMC11799391; doi:10.1038/s41598-025-88633-w)
Supplement: Supplementary file 1 — Supplementary Information 1. [file 41598_2025_88633_MOESM1_ESM.pdf]

SUPPLEMENTARY FIGURES FOR

Induced protein expression in *Leptospira* spp. and its application to CRISPR/Cas9 mutant generation

Fernandes, L.G.V.[1*]; Nascimento, A.L.T.O.[2]; Nally, J.E[1].

[1]Infectious Bacterial Diseases Research Unit, USDA Agricultural Research Service, National Animal Disease Center, Ames, IA, United States.

[2]Laboratório de Desenvolvimento de Vacinas, Instituto Butantan, São Paulo, Brazil.

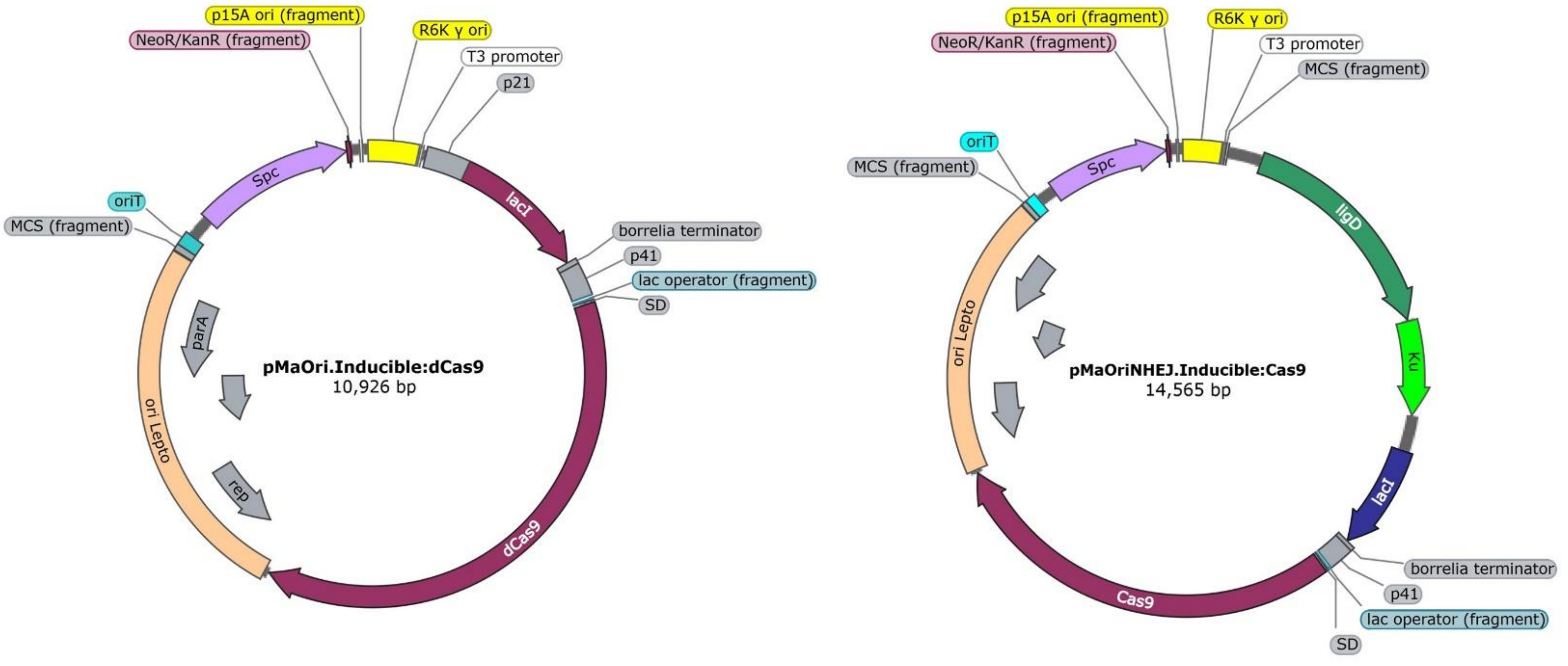

Supplementary Figure 1. Genetic map of plasmids for IPTG-controlled induction of dCas9 and Cas9

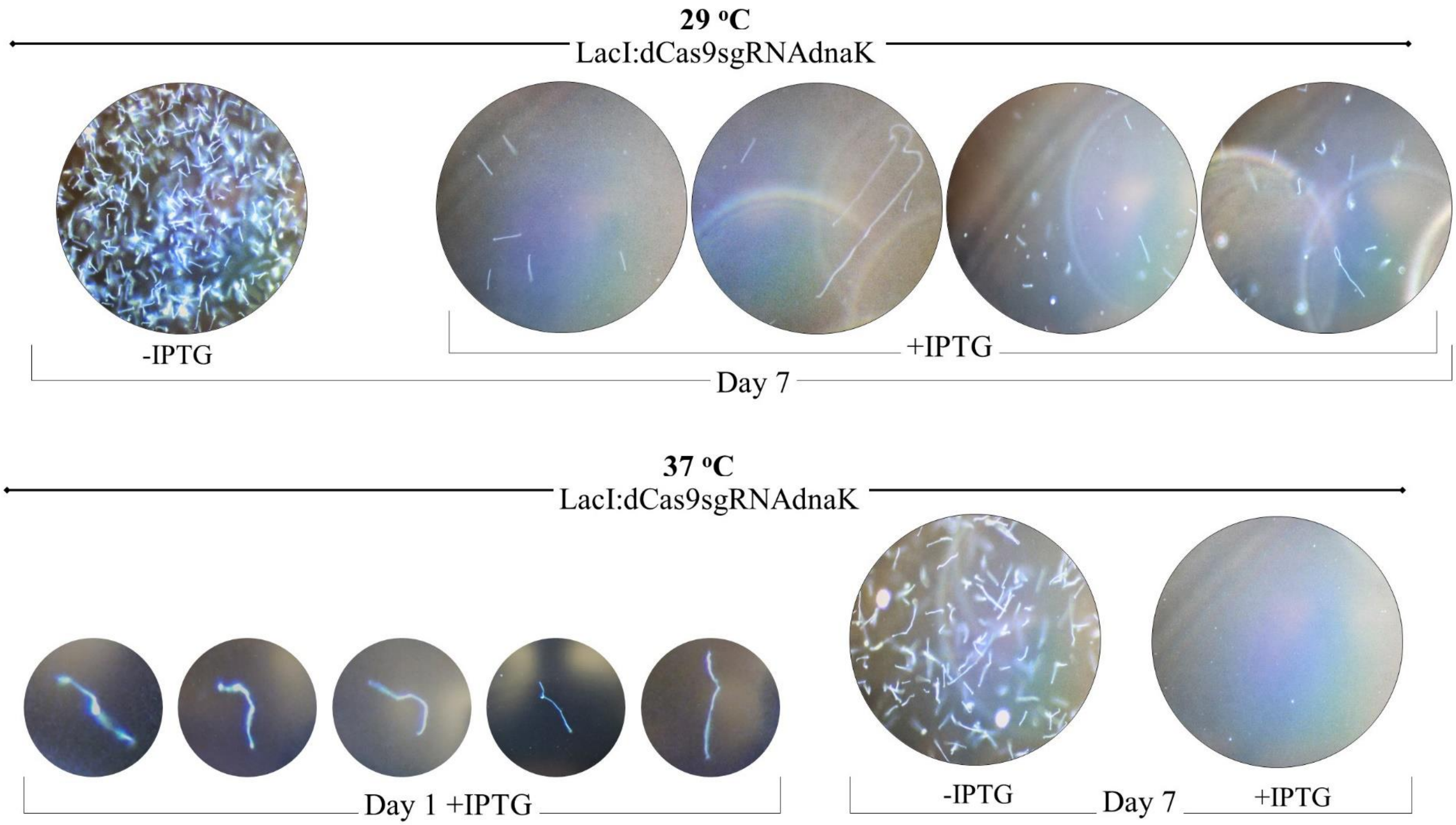

Supplementary Figure 2. Qualitative assessment of conditional *dnaK* knockdown mutants in HAN media at 29 and 37 ºC. *L. biflexa* cultures containing the plasmid for inducible silencing of the DnaK chaperone, either in the absence (-) or presence (+) of IPTG, were visualized by darkfield microscopy at either 29 ºC on day 7, or 37 ºC on day 1 and 7. Individual cells displaying abnormal morphology are shown (Day 1 + IPTG).

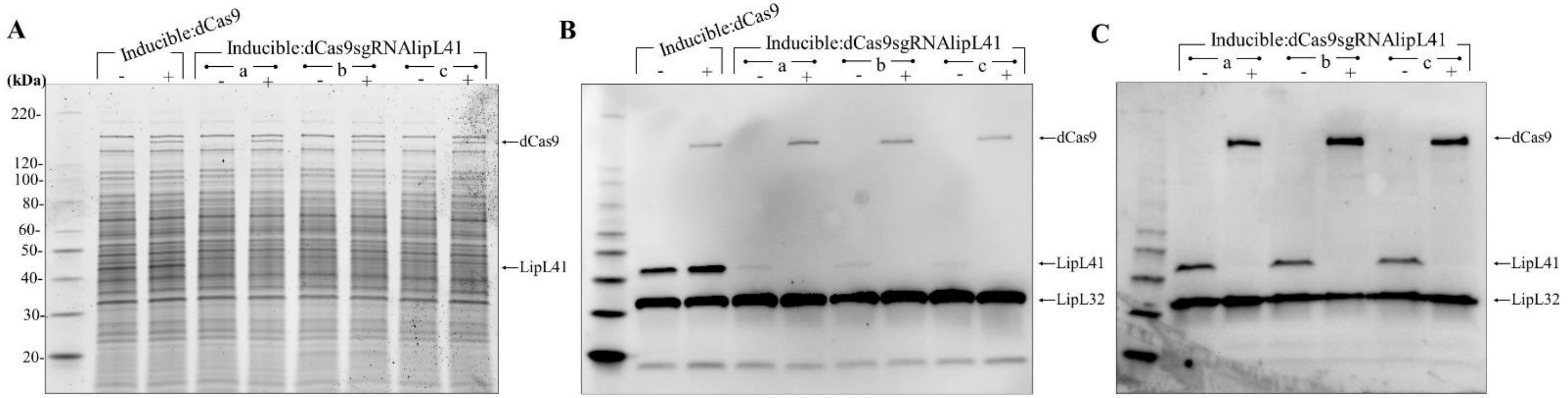

Supplementary Figure 3. Controlled silencing of LipL41 and confirmation of "leakage" of dCas9 expression. *L. interrogans* cells containing pMaOri.Inducible:dCas9 alone or with a sgRNA targeting *lipL41* gene were grown in liquid HAN with spectinomycin containing (+) or not (-) IPTG. Cell lysates were evaluated by Sypro Ruby **(A)** and immunoblotting **(B)** with anti-Cas9, anti-LipL41 and anti-LipL32 antibodies. **(C)** Clones containing the plasmid for silencing were re-evaluated by immunoblotting to better demonstrate the complete silencing of LipL41 in the presence of IPTG. However undetectable, dCas9 leakage is responsible for partial silencing of the target gene even when no IPTG is added to the media.
