# Supplementary material for: Induced protein expression in Leptospira spp. and its application to CRISPR/Cas9 mutant generation
Source: Sci Rep. 2025 Feb 5;15:4334. doi: 10.1038/s41598-025-88633-w (PMC11799391; doi:10.1038/s41598-025-88633-w)
Supplement: Supplementary file 2 — Supplementary Information 2. [file 41598_2025_88633_MOESM2_ESM.pdf]

Supplementary Table 1: Primers used in this study.

Primer	Sequence 5' – 3'	Purpose
pMaOriLipL41plasmid F	cggtggcggccgctctag	Amplify pMaOri backbone for *lipL41* ligation by Gibson.
pMaOriLipL41plasmid R	cggtggagctccagcttttg	
pMaOriLipL41gene F	gctggagctccaccgaacccaaaagttgttgattaattgt	Amplify *lipL41* promoter+gene for ligation into pMaOri by Gibson Assembly.
pMaOriLipL41gene R	gagcggccgccaccgcgcattcttcttcgttcgga	
pMaOriInducible F	tctggtttctttgaaattac	Amplify backbone+inducible cassette to substitute *lipL41* gene for *lipL32*.
InducibleCassette R	gaaaagtaacaccaatcctg	
LipL32 F	ttggtgttacttttcatgaaaaaactttcgattttgg	Amplify *lipL32* CDS for ligation with the inducible cassette.
LipL32 R	ttcaaagaaaccagattacttagtcgcgtcagaagc	
pdCas9 F	atggataagaaatactcaatagg	Amplification of plasmid pMaOri.dCas9 excluding the *S. pyogenes cas9* promoter.
pdCas9 R	caccgcggtggagctccagc	
cassette_dCas9 F	gctggagctccaccgcggtgagtggtatctactctcgcc	Amplification of inducible cassette for ligation into pdCas9-resulting amplicon.
cassette_dCas9 R	cctattgagtatttcttatccatgaaaagtaacaccaatcctg tttg	
pdCas9 F	atggataagaaatactcaatagg	Amplification of plasmid pMaOriCas9NHEJsmegmatis excluding the *S. pyogenes* cas9 promoter.
pNHEJ R	aaaagcttttttggctgc	
cassette_NHEJ F	gcagccaaaaaagcttttagtggtatctactctcgcc	Amplification of inducible cassette for ligation into pdCas9 F/pNHEJ R-resulting amplicon.
cassette_dCas9 R	cctattgagtatttcttatccatgaaaagtaacaccaatcctg tttg	
InducibleCassette F	agtggtatctactctcgcc	Amplification of whole inducible cassette for colony screening.
nducibleCassete R	gaaaagtaacaccaatcctg	
sgRNA F	ttaggatcccccggggaacaagaaagagtcagag	Amplification of sgRNA cassette and ligation by Gibson
sgRNA R	atcgaattcctgcagaaaaaagcaccgactcggtgc	

		assembly in a *Xma*I digested pMaOri backbone.
LipL21seqF	atgatgcaatccacatgtcgc	Amplification and sequencing of *lipL21* gene for assessing indel mutations.
LipL21seqR	ttattgtttggaaacctcttg	
RNaseP_sense F	tacttgagattgcacatcgggttttagagctagaaatagc	Substituting the protospacer for the one targeting the sense strand of RNase P
RNaseP_sense R	ccgatgtgcaatctcaagtagaaaatcacggtatgaacttagg	
RNaseP_antisense F	cgttatgagcctgtcccgccgttttagagctagaaatagc	Substituting the protospacer for the one targeting the antisense strand of RNase P
RNaseP_antisense R	ggcgggacaggctcataacggaaaatcacggtatgaacttagg	

Supplementary Table 2: Plasmid sequence

**pMaOri.Inducible:dCas9**
gtacgtactaagctctcatgtttcacgtactaagctctcatgtttaacgtactaagctctcat
gtttaacgaactaaaccctcatggctaacgtactaagctctcatggctaacgtactaagctct
catgtttcacgtactaagctctcatgtttgaacaataaaattaatataaatcagcaacttaaa
tagcctctaaggttttaagttttataagaaaaaaaagaatatataaggcttttaaagcttta
aggtttaacggttgtggacaacaagccagggatgtaacgcactgagaagcccttagagcctct
caaagcaattttgagtgacacaggaacacttaacggctgacatgggaattgcgcgcaattaac
cctcactaaagggaacaaaagctggagctccaccgcggtgagtggtatctactctcgccctta
gtaacaagtttgcataaaaagccccaaatttagaaatctcttgggatctgtcgtcgattcttt
ctaaagttacaggcaaccttccatccatttttccaggaagaaaagttatactcgtagaaaata
tcgggtgagtcaccaaacagaagaaaaaaaataaatttctcagcatgtagaacttagtatgat
ccaaatgaattcttagttttaatatgggaaatcttggattcaaagcaactcattaaaaaatgt
gttgcacaaactcggtaaagtattagctttttttcatttagtagatcaaggagaaaataatgaa
acctgttactctttacgacgtggcagaatatgctggtgtttcttatcaaactgtttcccgtgt
tgttaatcaagcgtcccatgtttctgcaaaaacccgtgaaaaagttgaagcagcaatggcgga
acttaactatatccctaatagagtagcacaacagcttgcgggaaaacaatctttacttattgg
agttgcgacttcttctcttgcattacatgcaccttctcaaattgttgcagcgattaaatccag
agcagatcaacttggagcttctgttgttgtttctatggttgaaagatctggtgtggaagcatg
taaagcagcagttcataatttacttgcgcaaagagtttccggacttatcatcaattatccgct
tgatgatcaagatgcgattgcagttgaagctgcttgtactaatgttcctgcactttttcttga
tgtgtctgatcaaaccccgatcaactctatcatcttttctcatgaagatggaacccgtcttgg
agttgaacatttagttgcattaggacatcaacaaatcgcattacttgcaggacctttatcttc
tgtatctgcgagattaagattagccggatggcataaatatcttacccgtaatcaaattcaacc
gatcgcagaacgtgaaggtgattggtctgcaatgtctggatttcaacaaaccatgcagatgct
taacgaaggtattgttcctactgcaatgcttgttgcgaatgatcaaatggcacttggagcaat
gagagcgattactgaatctggattaagagttggagcggatatttccgttgttggatatgatga
taccgaagattcctcctgttatatccctcctcttactaccatcaaacaggactttagacttct
tggacaaacctccgttgatcgtcttttacaattatctcaaggacaagcggtgaaaggtaatca
attacttcctgtgtctcttgttaaaagaaaaactacccttgcgcctaatactcaaactgcatc
tcctagagcattagcggattctttaatgcaattagcgagacaagtttccagacttgaatccgg
acaataactttttggctggctatattgcagccaaaaaaagcttttcgaacccaaaagttgtt
gattaattgtatcctcttctttttagaagtgacacaatttaaaagaataataatacaaaata
gaagaataaaaacaaattgtttgtgcacagcaaaacccatttgtaattcagtatcttgtatga
gaagtgtctctacaatcaaaaaaacaaagaacaaaaaatccgtttttcaaatcttagtttttc
gattctaaaataattgacatgattctttttgatttttaaatcatcccttatggaattgtgagc
ggataacaattttccccaaaatcaaacaggattggtgttacttttcatggataagaaatactc
aataggcttagctatcggcacaaatagcgtcggatgggcggtgatcactgatgaatataaggt
tccgtctaaaaagttcaaggttctgggaaatacagaccgccacagtatcaaaaaaaatcttat
aggggctcttttatttgacagtggagagacagcggaagcgactcgtctcaaacggacagctcg
tagaaggtatacacgtcggaagaatcgtatttgttatctacaggagatttttcaaatgagat
ggcgaaagtagatgatagtttctttcatcgacttgaagagtcttttttggtggaagaagacaa
gaagcatgaacgtcatcctatttttggaaatatagtagatgaagttgcttatcatgagaaata
tccaactatctatcatctgcgaaaaaaattggtagattctactgataaagcggatttgcgctt
aatctatttggccttagcgcatatgattaagtttcgtggtcatttttttgattgagggagattt

aaatcctgataatagtgatgtggacaaactatttatccagttggtacaaacctacaatcaatt
atttgaagaaaaccctattaacgcaagtggagtagatgctaaagcgattctttctgcacgatt
gagtaaatcaagacgattagaaaatctcattgctcagctccccggtgagaagaaaaatggctt
atttgggaatctcattgctttgtcattgggtttgaccccctaattttaaatcaaattttgattt
ggcagaagatgctaaattacagctttcaaaagatacttacgatgatgatttagataatttatt
ggcgcaaattggagatcaatatgctgatttgtttttggcagctaagaatttatcagatgctat
tttactttcagatatcctaagagtaaatactgaaataactaaggctcccctatcagcttcaat
gattaaacgctacgatgaacatcatcaagacttgactcttttaaaagctttagttcgacaaca
acttccagaaaagtataaagaaatctttttttgatcaatcaaaaaacggatatgcaggttatat
tgatgggggagctagccaagaagaattttataaatttatcaaaccaattttagaaaaaatgga
tggtactgaggaattattggtgaaactaaatcgtgaagatttgctgcgcaagcaacggacctt
tgacaacggctctattccccatcaaattcacttgggtgagctgcatgctattttgagaagaca
agaagacttttatccattttttaaaagacaatcgtgagaagattgaaaaaatcttgacttttcg
aattccttattatgttggtccattggcgcgtggcaatagtcgttttgcatggatgactcggaa
gtctgaagaaacaattaccccatggaattttgaagaagttgtcgataaaggtgcttcagctca
atcatttattgaacgcatgacaaactttgataaaaatcttccaaatgaaaaagtactaccaaa
acatagtttgctttatgagtattttacggtttataacgaattgacaaaggtcaaatatgttac
tgaaggaatgcgaaaaccagcatttctttcaggtgaacagaagaaagccattgttgatttact
cttcaaaacaaatcgaaaagtaaccgttaagcaattaaaagaagattatttcaaaaaaataga
atgttttgatagtgttgaaatttcaggagttgaagatagatttaatgcttcattaggtaccta
ccatgatttgctaaaaattattaaagataaagatttttttggataatgaagaaaatgaagatat
cttagaggatattgttttaacattgaccttatttgaagatagggagatgattgaggaaagact
taaaacatatgctcacctctttgatgataaggtgatgaaacagcttaaacgtcgccgttatac
tggttggggacgtttgtctcgaaaattgattaatggtattagggataagcaatctggcaaaac
aatattagatttttttgaaatcagatggttttgccaatcgcaattttatgcagctgatccatga
tgatagtttgacatttaaagaagacattcaaaaagcacaagtgtctggacaaggcgatagttt
acatgaacatattgcaaatttagctggtagccctgctattaaaaaaggtattttacagactgt
aaaagttgttgatgaattggtcaaagtaatggggcggcataagccagaaaatatcgttattga
aatggcacgtgaaaatcagacaactcaaaagggccagaaaaattcgcgagagcgtatgaaacg
aatcgaagaaggtatcaaagaattaggaagtcagattcttaaagagcatcctgttgaaaatac
tcaattgcaaaatgaaaagctctatctctattatctccaaaatggaagagacatgtatgtgga
ccaagaattagatattaatcgtttaagtgattatgatgtcgatgccattgttccacaaagttt
ccttaaagacgattcaatagacaataaggtcttaacgcgttctgataaaaatcgtggtaaatc
ggataacgttccaagtgaagaagtagtcaaaaagatgaaaaactattggagacaacttctaaa
cgccaagttaatcactcaacgtaagtttgataatttaacgaaagctgaacgtggaggtttgag
tgaacttgataaagctggttttatcaaacgccaattggttgaaactcgccaaatcactaagca
tgtggcacaaattttggatagtcgcatgaatactaaatacgatgaaaatgataaacttattcg
agaggttaaagtgattaccttaaaatctaaattagtttctgacttccgaaaagatttccaatt
ctataaagtacgtgagattaacaattaccatcatgcccatgatgcgtatctaaatgccgtcgt
tggaactgctttgattaagaaatatccaaaacttgaatcggagtttgtctatggtgattataa
agtttatgatgttcgtaaaatgattgctaagtctgagcaagaaataggcaaagcaaccgcaaa
atatttcttttactctaatatcatgaacttcttcaaaacagaaattacacttgcaaatggaga
gattcgcaaacgccctctaatcgaaactaatggggaaactggagaaattgtctgggataaagg
gcgagattttgccacagtgcgcaaagtattgtccatgccccaagtcaatattgtcaagaaaac
agaagtacagacaggcggattctccaaggagtcaattttaccaaaaagaaattcggacaagct
tattgctcgtaaaaaagactgggatccaaaaaaatatggtggttttgatagtccaacggtagc
ttattcagtcctagtggttgctaaggtggaaaaagggaaatcgaagaagttaaaatccgttaa

agagttactagggatcacaattatggaaagaagttcctttgaaaaaaatccgattgactttttt
agaagctaaaggatataaggaagttaaaaaagacttaatcattaaactacctaaatatagtct
ttttgagttagaaaacggtcgtaaacggatgctggctagtgccggagaattacaaaaaggaaa
tgagctggctctgccaagcaaatatgtgaattttttatatttagctagtcattatgaaaagtt
gaagggtagtccagaagataacgaacaaaaacaattgtttgtggagcagcataagcattattt
agatgagattattgagcaaatcagtgaattttctaagcgtgttattttagcagatgccaattt
agataaagttcttagtgcatataacaaacatagagacaaaccaatacgtgaacaagcagaaaa
tattattcatttatttacgtcgacgaatcttggagctcccgctgcttttaaatattttgatac
aacaattgatcgtaaacgatatacgtctacaaaagaagttttagatgccactcttatccatca
atccatcactggtctttatgaaacacgcattgatttgagtcagctaggaggtgactgaagtat
actttagatgaagattatttcttaatctagaaatccgtatagcatattcccacaaatgtggac
agaatatatgtggaataatctgacttttgacagaagtcgaaacacattccccacaaatgtggg
atgttcctcctgagtagatagtcacccaggaggaacatttttttatctccaatgccaaccta
tcccctacctactccagaaaatgggacggcaattaggccgccctactctgttcatcagaattt
ttaacaaaaacaacttcaattggtttctcgatttcttcgatgaagtattttggatgaccata
cctaacgaatctgaaactggttcgagtagtttcaattcggttccattgaaatctattttaatg
tttcttaatatatcattgctcgatcttgtgagtcgttcgttggcccagtcaaggaatccgatc
caagttggtttttgatttttttgcggcgtctacatttaatgttttttttactttcttctttagta
cgtacagacgcagaacgatagtcccactctgccctaccctgccgtacctccctaccttgacca
ttcgtcctagtctgactaggattattagctgatgcagttttggatattttatattgagcacgt
tccatcacgaccatatcgtgcaatgttgagcgaattaccctaacccgtccgtcgaaggaaatt
tgttcgatataccccgccttgcgcaatcgtgaaatatatttgcaattgttgtcgtggccatt
ccgagcattgtagcgagatattcgtttcccgcatagcagccgcccctcccctcgcaacgccct
gcaatatcgagtaacgtgattttagcgaggacgtctctaatcccccgtgaaagttttgtgttg
ataacagcacgaggtatgaactccccgtagatcaattcgtccatgatcgtttccccttttac
ggtgccggacatagcgcgggcgcagcacggtaaaagttttaccctactctaatatcggggaac
gcatagcattggcgtggagcctgtcggatggtcctcgattgtcgtcatggcctaatgttagag
cggccagtcttagttatgtcgcctaagataactatgactagaatcaggtacgatacaaatgtc
aagcacaaaatgagacatagttctattattgggatatttaggaaggatgcccttggggggctct
aacctccccgcgggctttgcgacgacaatctcggatgagatgatctattcggtactctaaaac
aggtgtacgaatttgggtaccatttttttgaaatttcgttaataaaaatatttaatcttacaaa
gagttaagatcatcatcaattgctgttatttgcctttttgcgtttgaaagaatttttttctttt
ttagtaattagatattcttttaggacacctttctcccgttgtgccaaccgagatccagattta
aatttctgataaatctcctttggatttagagtattgtcgcttgggtttaattctttttcttcg
gatacgggacctgctattaatttgtaagcgtcttccaaatgcgtcagtgttgacgcatttatc
agatcttttttttcgaatatttttatataccgttgaacggtgcgtattttaaaattgagattc
tcttcaacccatgggataaactctccatgtttaagttcgttttttttaacgaatagtatttcg
ccggcttcgatcgcgaagcgaaccatgtttttccggccgttaaaatcgattggtgtagtcgg
ttcagttcatccgcagttttatcgatcgttgacatgggtaattctgattctctttcgaatccc
gttcgccgtccgagtattgcggcctttttcttttcgctattcatttcgagagttcccttgcta
actcaagaaattgaatggctgcgtttgagttcgatttaatccgagtccccaatgtgaccgcgt
tctctaaggattcgcttctcgatatatgtgttttggttattggaatggtgtctccaatttcta
tgatcccctctgatttttttaggtgaaacattggaagggacacaaacgagttctgtattttac
ccgctctcctgtttgtcaaaatcatctcatctaacatctcgtaaccttgtagcgaccatcttg
aatatgaaattgggcagataacgagatcagaaacataaagacttgcgcgcagctcataacagt
cagcaggcggactgtcgataatcacataatcgtaatcaagagaacgaagtgatttttcaaata
acaacactgatccaaaatcattatggagttcagaatttacgctcgccaactttggagtagcag

gaatgcaatcaacttggagcgctgttgaataaacaacttcttcgatgttgattcgccccgtta
gggtctgccgaatatttttagatgaaatcgtttcgatagaaatgtctctaagaaaataatctg
ttaaattattgtttgggtccatgtcgattaccaaaactttaaatcccagtagagatagagctt
gagcaagaaatgcgtcgattgttgtttttccaacgccacccttcattgaggcaatacagtaaa
ttttcatttattgaaaacgttgcagggttgaatttttaggaaagcgtaatttttttttaggcgc
tgcgaagttgcaaaaatgagacatatttctgttattggatcgtaactgaatgtagagtaaatt
actgtttacaaggcgcctccttttgatactgtacgcaatgtatcgataccgacagattttatt
aacaaaattgtcgtagtaaatttaccaatgtaggcgataaatagagagagttccgtctaaatt
gatgagggtaggggattgcagtatgctgattaggatcccccgggctgcaggaattcgatatca
agcttatcgataccgtcgacgccggccagcctcgcagagcaggattcccgttgagcaccgcca
ggtgcgaataagggacagtgaagaaggaacacccgctcgcgggtgggcctacttcacctatcc
tgcccggctgacgccgttggatacaccaaggaaagtctacacgaaccctttggcaaaatcctg
tatatcgtgcgaaaaaggatggatataccgaaaaaatcgctataatgaccccgaagcagggtt
atgcagcggaaaagcgctggtacccaattcgccctatagtgagtcgtagggggcccgagcttc
aaggaagatttcctattaaggttgaacttaagagcttaagcatagacgatttgaaaaaaattt
taaaacaaacaaaaaattctttaataaaacaatatgttgcgatgtttaaggtttatgatttag
atttaaagtttagtgaggaagctatagatagaattgcagagcttacttttaatatgaatcttg
agagtgaaaatcttggtgccagaagacttcacggtgttatggaaatagtgcttgcagatcttt
tttttgaagtgcctggcagtaagttgaaaaaatttgaaataaacttggactatgttaataaaa
aaatacaaattaacgaacaaaaagatttgaactattatataatttagttaaaagcaattttaa
atgagggaggtttccatatgagcaatttgattaacggaaaaataccaaatcaagcgattcaaa
cattaaaaatcgtaaaagatttgtttggaagttcaatagttggagtatatctatttggttcag
cagtaaatggtggtttacgcattaacagcgatgtagatgttctagtcgtcgtgaatcatagtt
tacctcaattaactcgaaaaaaactaacagaaagactaatgactatatcaggaaagattggaa
atacggattctgttagaccacttgaagttacggttataaataggagtgaagttgtcccttggc
aatatcctccaaaaagagaatttatatacggtgagtggctcaggggtgaatttgagaatggac
aaattcaggaaccaagctatgatcctgatttggctattgttttagcacaagcaagaaagaata
gtatttctctatttggtcctgattcttcaagtatacttgtctccgtacctttgacagatattc
gaagagcaattaaggattctttgccagaactaattgaggggataaaaggtgatgagcgtaatg
taattttaaccctagctcgaatgtggcaaacagtgactactggtgaaattacctcgaaagatg
tcgctgcagaatgggctatacctcttttacctaaagagcatgtaactttactggatatagcta
gaaaaggctatcggggagagtgtgatgataagtgggaaggactatattcaaaggtgaaagcac
tcgttaagtatatgaaaaattctatagaaacttctctcaattaggctaattttattgcaataa
caggtgcttactttccccctcgccttctatcgccttcttgacgagttcttctgactgcagttt
ttttaaggcagttattggtgcctagaaatattttatctgattaataagatgatcttcttgaga
tcgttttggtctgcgacgatatcagatctgatccggccacgatgcgtccggcgtagaggatct
gaagatcagcagttcaacctgttgata

pMaOriNHEJ.Inducible:Cas9

gtacgtactaagctctcatgtttcacgtactaagctctcatgtttaacgtactaagctctcat
gtttaacgaactaaaccctcatggctaacgtactaagctctcatggctaacgtactaagctct
catgtttcacgtactaagctctcatgtttgaacaataaaattaatataaatcagcaacttaaa
tagcctctaaggttttaagttttataagaaaaaaaagaatatataaggcttttaaagctttta
aggtttaacggttgtggacaacaagccagggatgtaacgcactgagaagcccttagagcctct
caaagcaattttgagtgacacaggaacacttaacggctgacatgggaattgcgcgcaattaac
cctcactaaagggaacaaaagctggagctccaccgcggtggcggccgaacaagaaagagtcag
agaattattgaagagatactcttatactaccgtctttggaagaattttcgcatggattttaga

```
tttgctggactggttgaagcgatttttcaaaaaaaataatcaatttgtgtctgagatttgaa
aacgcttgtttgatagtttttaagaatttctgatgtttcaatcgtatagaaattctaaattt
agaaatcatcctttacttttctctaagacttatataacaatcgctttaaactcaaattataat
ctttcagataaaaaattattcaatattgatttacaaaaaattcctaagttcataccgtgattt
tcctaactaaggagagtctatggaacgttatgaaagagtgagacttacgaacccggacaaagt
tttatatcctgcaactggaactaccaaagcggaagtttttgactactatctttctatcgcgca
agttatgttacctcatattgcaggtagaccggttactagaaaaagatggcctaatggtgttgc
agaagaagcctttttttgaaaaacaacttgcttcttccgcaccgtcttggttagaaagaggatc
tattactcataaatctggaaccaccacctatccgatcatcaatactagagaaggattagcatg
gattgcacaacaagcatctcttgaagttcatgttcctcaatggcgttttgaagatggtgatca
aggacctgcaactcgtattgtttttgatttagatcctggtgaaggtgtgactatgactcaatt
atgtgaagttgcgcatgaagtgagagaacttatgtctgatcttgaattgcaaacctatcctct
tacctctggatctaaaggacttcatctttatgttcctcttgcgcaacctatttcttccagagg
tgcttctgttttagcaagaagagttgctcaacaacttgaacaagcaatgcctaaacttgttac
tgcgactatgaccaaatctcttagagcgggaaaagtttttcttgactggtcccaaaataatgc
agcgaaaactactattgcgccttattctttaagaggaagagatcatcctactgttgcagcacc
tagaacttgggatgaaatctctgatcctgaacttcgtcatcttcgttttgatgaagttcttga
tcgtcttgacgaatacggtgatttattagcacctttagatgcggatgctcctcttgcagataa
actttctacttatagatccatgcgtgacgcgtctaaaactcctgaacctgttcctaaagaaat
ccctactaccggaaacaatgataaatttgttattcaagaacatcatgcgcgtcgacttcatta
tgatcttagattagaacgtgacggtgtgcttgtttcttttgcagttcctaaaatcttcctga
aactactgcggaaaatcgtcttgcagttcatactgaagatcatccgatcgaatatcttacctt
tcacggatctattccgaaaggtgaatatggtgctggtgatatggttatttgggatgcgggaac
atatgaaactgaaaaatttcgtgttcctgaagaacttgataaccctgatgatactcatggtga
aatcatcgttacccttcacggtgaaaaagttgatggaagatatgcgcttatccaaactaaagg
taaaaactggcttgcgcacagaatgaaagatcaaaaaaacacccgtcctgaagatttgcacc
tatgttagcaactgaaggatccgtgcaaaaatacaaagcaactcaatgggcctttgaaggtaa
atgggatggatatagagttatcgttcatgtggatcatggaaaacttcaaatcagatccagaac
cggaagagatgttacaagagaataccctcaatttaaagcacttgcagcagatttagcggatca
tcatgttgttttagatggtgaagcagttgcgcttgataataatggaattccgtcttttggaga
aatgcaaaacagagcgagatctaccagagttgaattttgggcgtttgatatcttatggcttga
tggacgttctcttcttcgtgcgaaatattccgatagaagaaaaatcttagaagcgcttgcaga
tggtggatctttaattgttcctgatcaactttctggtgatggacctgaagcaatggaacatgc
aagagaaaaaagatacgaaggtgtggttgcgaaaaaacgtgattctacatatcaaccgggaag
aagatctagttcttggattaaagacaaaatttggaatactcaagaagttgttatcggaggttg
gagacaaggtgaaggtggaagatcctctggaattggagctttagttttaggaattccaggacc
tgatggacttcaatttgttggaagagttggaactggatttaccgaaaaagaacttggaaaatt
gaaagatatgcttaaaccgcttcacaccgatgaatctccatttgatgcacctttacctaaagt
agatgcacgtggtgttacttttgttagacctgaattagttggagaagtgcgttattctgaaag
aacttctgacggaagattgagacaaccatcttggagaggattaagacctgataaaactccgga
tgaagttgtgtgggaataacctaactaaggagagtctatgagatccatctggaaaggatctat
tgcgtttggacttgttaacgttccggttaaagtatattctgcgaccgaagatcacgatatcaa
atttcatcaagttcatgcgaaagataatggaagaatccgttacaaacgtgtttgtgaagtttg
tggtgaagtggttgaatatagagacatcaataaagcgtttgaatccgacgatggacaaatggt
tgttatcactgatgaagatattgcgaccttgcctgaagaaagatctcgtgaaatcgaagtggt
agaatttattcctgcggaacaattggatccgcttatgtatgataaatcttatttcttgaacc
ggattctaaatcttccaaatcctatgtgcttttggcgaaaactttagcggaaactgatagaat
```

```
tgcgatcgttcatttttcccttcgtaacaaatccagattagcagcgcttcgtgttaaagactt
ttccaaacgtgatgttatgatgattcacaccttactttggcctgatgaaatcagagatcctga
ctttcctatccttgacaaagaagttcaaatcaaacctgcagaacttaaaatggcaggacaagt
tgtagaatccatgaccgatgattttaaaccggatctttatcacgacgactaccaagaacaact
tcgtgaattagtacaggcgaaattagaaggtggtgaagctttttctgttgaagaacaaccagc
ggaattagacgaaggtacagaagatgtttctgaccttttagcaaaacttgaagcgtctgttaa
agcgcgtaaaggtggaaaatctgattccaaagatgattccgattctgaatccgattctaaaga
atctaaatccgactccaaaccggcaaaaaaagctcctgctaaaaaagcagcagcaatgaaatc
tacagcgaaaaaagcgccagcaaaaaaagctgcggcaaaaaaatcttaactttttggctggc
tatattgcagccaaaaaaagcttttagtggtatctactctcgcccttagtaacaagtttgcat
aaaaagccccaaatttagaaatctcttgggatctgtcgtcgattctttctaaagttacaggca
accttccatccatttttccaggaagaaaagttatactcgtagaaaatatcgggtgagtcacca
aacagaagaaaaaaaataaatttctcagcatgtagaacttagtatgatccaaatgaattctta
gttttaatatgggaaatcttggattcaaagcaactcattaaaaaatgtgttgcacaaactcgg
taaagtattagctttttttcatttagtagatcaaggagaaaataatgaaacctgttactcttta
cgacgtggcagaatatgctggtgtttcttatcaaactgtttcccgtgttgttaatcaagcgtc
ccatgtttctgcaaaaacccgtgaaaaagttgaagcagcaatggcggaacttaactatatccc
taatagagtagcacaacagcttgcgggaaaacaatctttacttattggagttgcgacttcttc
tcttgcattacatgcaccttctcaaattgttgcagcgattaaatccagagcagatcaacttgg
agcttctgttgttgtttctatggttgaaagatctggtgtggaagcatgtaaagcagcagttca
taatttacttgcgcaaagagtttccggacttatcatcaattatccgcttgatgatcaagatgc
gattgcagttgaagctgcttgtactaatgttcctgcactttttcttgatgtgtctgatcaaac
cccgatcaactctatcatcttttctcatgaagatggaacccgtcttggagttgaacatttagt
tgcattaggacatcaacaaatcgcattacttgcaggacctttatcttctgtatctgcgagatt
aagattagccggatggcataaatatcttacccgtaatcaaattcaaccgatcgcagaacgtga
aggtgattggtctgcaatgtctggatttcaacaaaccatgcagatgcttaacgaaggtattgt
tcctactgcaatgcttgttgcgaatgatcaaatggcacttggagcaatgagagcgattactga
atctggattaagagttggagcggatatttccgttgttggatatgatgataccgaagattcctc
ctgttatatccctcctcttactaccatcaaacaggactttagacttcttggacaaacctccgt
tgatcgtcttttacaattatctcaaggacaagcggtgaaaggtaatcaattacttcctgtgtc
tcttgttaaaagaaaaactacccttgcgcctaatactcaaactgcatctcctagagcattagc
ggattctttaatgcaattagcgagacaagtttccagacttgaatccggacaataactttttg
gctggctatattgcagccaaaaaaagcttttcgaacccaaaagttgttgattaattgtatcct
cttctttttagaagtgacacaatttaaaagaataataatacaaaatagaagaataaaaacaa
attgtttgtgcacagcaaaacccatttgtaattcagtatcttgtatgagaagtgtctctacaa
tcaaaaaaacaaagaacaaaaaatccgtttttcaaatcttagtttttcgattctaaaataatt
gacatgattctttttgatttttaaatcatcccttatggaattgtgagcggataacaattttcc
ccaaaatcaaacaggattggtgttacttttcatggataagaaatactcaataggcttagatat
cggcacaaatagcgtcggatgggcggtgatcactgatgaatataaggttccgtctaaaaagtt
caaggttctgggaaatacagaccgccacagtatcaaaaaaaatcttataggggctcttttatt
tgacagtggagagacagcggaagcgactcgtctcaaacggacagctcgtagaaggtatacacg
tcggaagaatcgtatttgttatctacaggagatttttcaaatgagatggcgaaagtagatga
tagtttctttcatcgacttgaagagtctttttggtggaagaagacaagaagcatgaacgtca
tcctattttggaaatatagtagatgaagttgcttatcatgagaaatatccaactatctatca
tctgcgaaaaaaattggtagattctactgataaagcggatttgcgcttaatctatttggcctt
agcgcatatgattaagtttcgtggtcatttttttgattgagggagatttaaatcctgataatag
tgatgtggacaaactatttatccagttggtacaaacctacaatcaattatttgaagaaaccc
```

tattaacgcaagtggagtagatgctaaagcgattctttctgcacgattgagtaaatcaagacg
attagaaaatctcattgctcagctccccggtgagaagaaaaatggcttatttgggaatctcat
tgctttgtcattgggtttgacccctaattttaaatcaaattttgatttggcagaagatgctaa
attacagctttcaaaagatacttacgatgatgatttagataatttattggcgcaaattggaga
tcaatatgctgatttgtttttggcagctaagaatttatcagatgctattttactttcagatat
cctaagagtaaatactgaaataactaaggctcccctatcagcttcaatgattaaacgctacga
tgaacatcatcaagacttgactcttttaaaagctttagttcgacaacaacttccagaaaagta
taaagaaatctttttttgatcaatcaaaaaacggatatgcaggttatattgatggggggagctag
ccaagaagaattttataaatttatcaaaccaattttagaaaaaatggatggtactgaggaatt
attggtgaaactaaatcgtgaagatttgctgcgcaagcaacggacctttgacaacggctctat
tccccatcaaattcacttgggtgagctgcatgctattttgagaagacaagaagacttttatcc
atttttaaaagacaatcgtgagaagattgaaaaaatcttgacttttcgaattccttattatgt
tggtccattggcgcgtggcaatagtcgttttgcatggatgactcggaagtctgaagaaacaat
taccccatggaattttgaagaagttgtcgataaaggtgcttcagctcaatcatttattgaacg
catgacaaactttgataaaaatcttccaaatgaaaaagtactaccaaaacatagtttgcttta
tgagtattttacggtttataacgaattgacaaaggtcaaatatgttactgaaggaatgcgaaa
accagcatttctttcaggtgaacagaagaaagccattgttgatttactcttcaaaacaaatcg
aaaagtaaccgttaagcaattaaaagaagattatttcaaaaaaatagaatgttttgatagtgt
tgaaatttcaggagttgaagatagatttaatgcttcattaggtacctaccatgatttgctaaa
aattattaaagataaagatttttttggataatgaagaaaatgaagatatcttagaggatattgt
tttaacattgaccttatttgaagatagggagatgattgaggaaagacttaaaacatatgctca
cctctttgatgataaggtgatgaaacagcttaaacgtcgccgttatactggttggggacgttt
gtctcgaaaattgattaatggtattagggataagcaatctggcaaaacaatattagattttt
gaaatcagatggttttgccaatcgcaattttatgcagctgatccatgatgatagtttgacatt
taaagaagacattcaaaaagcacaagtgtctggacaaggcgatagtttacatgaacatattgc
aaatttagctggtagccctgctattaaaaaaggtattttacagactgtaaaagttgttgatga
attggtcaaagtaatggggcggcataagccagaaaatatcgttattgaaatggcacgtgaaaa
tcagacaactcaaaagggccagaaaaattcgcgagagcgtatgaaacgaatcgaagaaggtat
caaagaattaggaagtcagattcttaaagagcatcctgttgaaaatactcaattgcaaaatga
aaagctctatctctattatctccaaaatggaagagacatgtatgtggaccaagaattagatat
taatcgtttaagtgattatgatgtcgatcacattgttccacaaagtttccttaaagacgattc
aatagacaataaggtcttaacgcgttctgataaaaatcgtggtaaatcggataacgttccaag
tgaagaagtagtcaaaaagatgaaaaactattggagacaacttctaaacgccaagttaatcac
tcaacgtaagtttgataatttaacgaaagctgaacgtggaggtttgagtgaacttgataaagc
tggttttatcaaacgccaattggttgaaactcgccaaatcactaagcatgtggcacaaatttt
ggatagtcgcatgaatactaaatacgatgaaaatgataaacttattcgagaggttaaagtgat
taccttaaaatctaaattagtttctgacttccgaaaagatttccaattctataaagtacgtga
gattaacaattaccatcatgcccatgatgcgtatctaaatgccgtcgttggaactgctttgat
taagaaatatccaaaacttgaatcggagtttgtctatggtgattataaagtttatgatgttcg
taaaatgattgctaagtctgagcaagaaataggcaaagcaaccgcaaaatatttcttttactc
taatatcatgaacttcttcaaaacagaaattacacttgcaaatggagagattcgcaaacgccc
tctaatcgaaactaatggggaaactggagaaattgtctgggataaagggcgagattttgccac
agtgcgcaaagtattgtccatgccccaagtcaatattgtcaagaaaacagaagtacagacagg
cggattctccaaggagtcaattttaccaaaaagaaattcggacaagcttattgctcgtaaaaa
agactgggatccaaaaaaatatggtggttttgatagtccaacggtagcttattcagtcctagt
ggttgctaaggtggaaaaagggaaatcgaagaagttaaaatccgttaaagagttactagggat
cacaattatggaaagaagttcctttgaaaaaaatccgattgacttttttagaagctaaaggata

```
taaggaagttaaaaaagacttaatcattaaactacctaaatatagtcttttgagttagaaaa
cggtcgtaaacggatgctggctagtgccggagaattacaaaaaggaaatgagctggctctgcc
aagcaaatatgtgaatttttatatttagctagtcattatgaaaagttgaagggtagtccaga
agataacgaacaaaaacaattgtttgtggagcagcataagcattatttagatgagattattga
gcaaatcagtgaattttctaagcgtgttattttagcagatgccaatttagataaagttcttag
tgcatataacaaacatagagacaaaccaatacgtgaacaagcagaaaatattattcatttatt
tacgttgacgaatcttggagctcccgctgcttttaaatattttgatacaacaattgatcgtaa
acgatatacgtctacaaaagaagttttagatgccactcttatccatcaatccatcactggtct
ttatgaaacacgcattgatttgagtcagctaggaggtgactgaagtatattctagatgaagat
tatttcttaatctagaaatccgtatagcatattcccacaaatgtggacagaatatatgtggaa
taatctgacttttgacagaagtcgaaacacattccccacaaatgtgggatgttcctcctgagt
agatagtcacccaggaggaacatttttttatctccaatgccaacctatcccctacctactcc
agaaaatgggacggcaattaggccgccctactctgttcatcagaattttaacaaaaacaact
tcaattggtttctcgatttcttcgatgaagtattttggatgaccatacctaacgaatctgaa
actggttcgagtagtttcaattcggttccattgaaatctattttaatgtttcttaatatatca
ttgctcgatcttgtgagtcgttcgttggcccagtcaaggaatccgatccaagttggttttga
tttttgcggcgtctacatttaatgtttttttactttcttctttagtacgtacagacgcagaa
cgatagtcccactctgccctaccctgccgtacctccctaccttgaccattcgtcctagtctga
ctaggattattagctgatgcagttttggatattttatattgagcacgttccatcacgaccata
tcgtgcaatgttgagcgaattaccctaacccgtccgtcgaaggaaatttgttcgatatacccc
gccttgcgcaatcgtgaaatatatttgcaattgttgtcgtggccattccgagcattgtagcg
agatattcgtttcccgcatagcagccgcccctcccctcgcaacgccctgcaatatcgagtaac
gtgattttagcgaggacgtctctaatccccgtgaaagttttgtgttgataacagcacgaggt
atgaactccccgtagatcaattcgtccatgatcgtttccccttttacggtgccggacatagc
gcgggcgcagcacggtaaaagttttaccctactctaatatcggggaacgcatagcattggcgt
ggagcctgtcggatggtcctcgattgtcgtcatggcctaatgttagagcggccagtcttagtt
atgtcgcctaagataactatgactagaatcaggtacgatacaaatgtcaagcacaaaatgaga
catagttctattattgggatatttaggaaggatgcccttgggggctctaacctccccgcgggc
tttgcgacgacaatctcggatgagatgatctattcggtactctaaaacaggtgtacgaatttg
ggtaccatttttgaaatttcgttaataaaaatatttaatcttacaaagagttaagatcatca
tcaattgctgttatttgccttttgcgtttgaaagaatttttctttttttagtaattagatat
tcttttaggacacctttctcccgttgtgccaaccgagatccagatttaaatttctgataaatc
tcctttggatttagagtattgtcgcttgggtttaattctttttcttcggatacggacctgct
attaatttgtaagcgtcttccaaatgcgtcagtgttgacgcatttatcagatctttttttttcg
aatattttatataccgttgaacggtgcgtattttaaaattgagattctcttcaacccatggg
ataaactctccatgtttaagttcgtttttttttaacgaatagtatttcgccggcttcgatcgcg
aagcgaaccatgtttttttccggccgttaaaatcgattggtgtagtcggttcagttcatccgca
gttttatcgatcgttgacatgggtaattctgattctctttcgaatcccgttcgccgtccgagt
attgcggcctttttcttttcgctattcatttcgagagttcccttgctaactcaagaaattgaa
tggctgcgtttgagttcgatttaatccgagtccccaatgtgaccgcgttctctaaggattcgc
ttctcgatatatgtgttttggttattggaatggtgtctccaatttctatgatcccctctgatt
ttttaggtgaaacattggaagggacacaaacgagttctgtatttttacccgctctcctgtttg
tcaaaatcatctcatctaacatctcgtaaccttgtagcgaccatcttgaatatgaaattgggc
agataacgagatcagaaacataaagacttgcgcgcagctcataacagtcagcaggcggactgt
cgataatcacataatcgtaatcaagagaacgaagtgatttttcaaataacaacactgatccaa
aatcattatggagttcagaatttacgctcgccaactttggagtagcaggaatgcaatcaactt
ggagcgctgttgaataaacaacttcttcgatgttgattcgccccgttagggtctgccgaatat
```

ttttagatgaaatcgtttcgatagaaatgtctctaagaaaataatctgttaaattattgtttg
ggtccatgtcgattaccaaaactttaaatcccagtagagatagagcttgagcaagaaatgcgt
cgattgttgtttttccaacgccacccttcattgaggcaatacagtaaattttcatttattgaa
aacgttgcagggttgaattttaggaaagcgtaatttttttaggcgctgcgaagttgcaaaa
atgagacatatttctgttattggatcgtaactgaatgtagagtaaattactgtttacaaggcg
cctccttttgatactgtacgcaatgtatcgataccgacagattttattaacaaaattgtcgta
gtaaatttaccaatgtaggcgataaatagagagagttccgtctaaattgatgagggtaggga
ttgcagtatgctgattaggatcccccgggctgcaggaattcgatatcaagcttatcgataccg
tcgacgccggccagcctcgcagagcaggattcccgttgagcaccgccaggtgcgaataaggga
cagtgaagaaggaacacccgctcgcgggtgggcctacttcacctatcctgcccggctgacgcc
gttggatacaccaaggaaagtctacacgaaccctttggcaaaatcctgtatatcgtgcgaaaa
aggatggatataccgaaaaaatcgctataatgaccccgaagcagggttatgcagcggaaaagc
gctggtacccaattcgccctatagtgagtcgtagggggcccgagcttcaaggaagatttccta
ttaaggttgaacttaagagcttaagcatagacgatttgaaaaaaatttaaaacaaacaaaaa
attctttaataaaacaatatgttgcgatgtttaaggtttatgatttagatttaaagtttagtg
aggaagctatagatagaattgcagagcttacttttaatatgaatcttgagagtgaaaatcttg
gtgccagaagacttcacggtgttatggaaatagtgcttgcagatctttttttgaagtgcctg
gcagtaagttgaaaaaatttgaaataaacttggactatgttaataaaaaaatacaaattaacg
aacaaaaagatttgaactattatataatttagttaaaagcaattttaaatgagggaggtttcc
atatgagcaatttgattaacggaaaaataccaaatcaagcgattcaaacattaaaaatcgtaa
aagatttgtttggaagttcaatagttggagtatatctatttggttcagcagtaaatggtggtt
tacgcattaacagcgatgtagatgttctagtcgtcgtgaatcatagtttacctcaattaactc
gaaaaaaactaacagaaagactaatgactatatcaggaaagattggaaatacggattctgtta
gaccacttgaagttacggttataaataggagtgaagttgtcccttggcaatatcctccaaaaa
gagaatttatatacggtgagtggctcaggggtgaatttgagaatggacaaattcaggaaccaa
gctatgatcctgatttggctattgttttagcacaagcaagaaagaatagtatttctctatttg
gtcctgattcttcaagtatacttgtctccgtacctttgacagatattcgaagagcaattaagg
attctttgccagaactaattgaggggataaaaggtgatgagcgtaatgtaattttaaccctag
ctcgaatgtggcaaacagtgactactggtgaaattacctcgaaagatgtcgctgcagaatggg
ctatacctcttttacctaaagagcatgtaactttactggatatagctagaaaaggctatcggg
gagagtgtgatgataagtggaaggactatattcaaaggtgaaagcactcgttaagtatatga
aaaattctatagaaacttctctcaattaggctaattttattgcaataacaggtgcttactttc
cccctcgccttctatcgccttcttgacgagttcttctgactgcagtttttttaaggcagttat
tggtgcctagaaatatttatctgattaataagatgatcttcttgagatcgttttggtctgcg
acgatatcagatctgatccggccacgatgcgtccggcgtagaggatctgaagatcagcagttc
aacctgttgata
